# Bio-Chemoinformatics-Driven Analysis of nsp7 and nsp8 Mutations and Their Effects on Viral Replication Protein Complex Stability

**DOI:** 10.3390/cimb46030165

**Published:** 2024-03-18

**Authors:** Bryan John J. Subong, Takeaki Ozawa

**Affiliations:** Department of Chemistry, School of Science, University of Tokyo, 7-3-1 Hongo, Bunkyo-ku, Tokyo 113-8654, Japan; bjsubong@chem.s.u-tokyo.ac.jp

**Keywords:** bioinformatics, chemoinformatics, SARS-CoV-2, nsp7, nsp8, mutation

## Abstract

The nonstructural proteins 7 and 8 (nsp7 and nsp8) of SARS-CoV-2 are highly important proteins involved in the RNA-dependent polymerase (RdRp) protein replication complex. In this study, we analyzed the global mutation of nsp7 and nsp8 in 2022 and 2023 and analyzed the effects of mutation on the viral replication protein complex using bio-chemoinformatics. Frequently occurring variants are found to be single amino acid mutations for both nsp7 and nsp8. The most frequently occurring mutations for nsp7 which include L56F, L71F, S25L, M3I, D77N, V33I and T83I are predicted to cause destabilizing effects, whereas those in nsp8 are predicted to cause stabilizing effects, with the threonine to isoleucine mutation (T89I, T145I, T123I, T148I, T187I) being a frequent mutation. A conserved domain database analysis generated critical interaction residues for nsp7 (Lys-7, His-36 and Asn-37) and nsp8 (Lys-58, Pro-183 and Arg-190), which, according to thermodynamic calculations, are prone to destabilization. Trp-29, Phe-49 of nsp7 and Trp-154, Tyr-135 and Phe-15 of nsp8 cause greater destabilizing effects to the protein complex based on a computational alanine scan suggesting them as possible new target sites. This study provides an intensive analysis of the mutations of nsp7 and nsp8 and their possible implications for viral complex stability.

## 1. Introduction

SARS-CoV-2, or severe acute respiratory syndrome coronavirus 2, is the causative agent of the COVID-19 pandemic and is the seventh coronavirus known to infect humans [1]. As of 21 March 2023, the World Health Organization has reported 761,071,826 confirmed cases of COVID-19, with 6,879,677 deaths recorded [2,3]. The increasing number of viral infections has become a global problem that has caused tremendous harm to health [4] and economic impacts [5,6].

**Figure 1:**
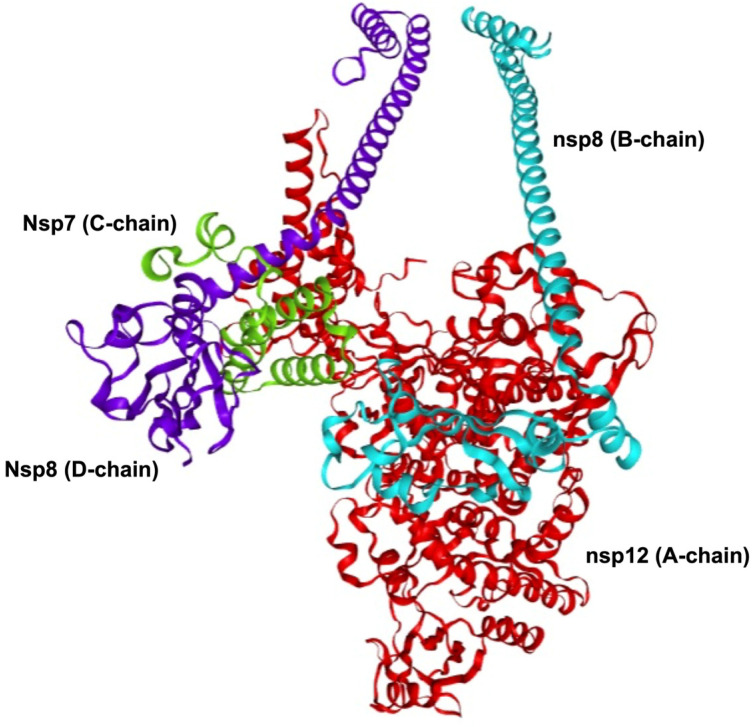
SARS-CoV-2 viral replication protein complex. The viral replication protein complex is primarily comprised of the nsp12–nsp7–nsp8 supercomplex. The nsp12 (A-chain, shown in red) is the main catalytic subunit of the protein complex. The nsp7 (C-chain, shown in green) functions as a cofactor that binds to nsp12. The nsp8 (B-chain, shown in cyan; D-chain, shown in purple) functions as a cofactor and as a helper in extending the template RNA-binding surface. This viral replication protein complex represents the minimal machinery of the virus that can perform nucleotide polymerization. The viral replication protein complex shown was modelled using Robetta comparative modelling using PDB: 8GWE as template.

The viral replication complex of SARS-CoV-2 is primarily composed of three nonstructural proteins: nonstructural protein 12 (nsp12), which comprises the A-chain; nonstructural protein 7 (nsp7), which comprises the C-chain; and nonstructural protein 8 (nsp8), which comprises the B- and D-chains [7] (Figure 1). Cryo-EM studies have shown that the SARS-CoV-2 polymerase complex comprises an nsp12 core subunit bound to an nsp7-8 heterodimer and another nsp8 monomer bound to the complex at a site different from that of nsp7-8 [7,8]. These three proteins form the nsp12–nsp7–nsp8 supercomplex of the viral replication protein complex. This viral replication protein complex represents the minimal machinery of the virus that can perform nucleotide polymerization [9]. The viral replication protein complex regulates the replication of the SARS-CoV-2 genome, highlighting its significance in the viral life cycle [10]. This has been further studied as a hotspot for drug targeting, emphasizing the importance of understanding the structure and mechanism of this dynamic assembly [3,11].

Nsp12, a 106-kDa protein, is the main catalytic subunit of the protein complex [12,13]. Its structure comprises an N-terminal nidovirus RNA-dependent RNA polymerase (RdRp)-associated nucleotidyltransferase and a C-terminal right-hand RdRp domain [13]. Owing to its essential role in viral replication, nsp12 has been a primary target for antiviral drug development, such as remdesivir, which is an inhibitor of RdRp polymerases [14], and biologics, such as monoclonal antibodies [15].

The other two proteins, nsp7 and nsp8, have recently been gaining attention owing to their essential role in the viral replication process, since nsp12 alone possesses little activity and requires nsp7 and nsp8 for RNA synthesis activity [16].

Nsp7, a 9-kDa protein that is primarily alpha-helical in structure, functions as a cofactor that binds to nsp12, allowing stabilization of the polymerase domain [17]. Nsp8, a 24-kDa protein comprising both alpha-helical and beta-strands, functions as a co-factor for nsp12 binding and is critically important in extending the template RNA-binding surface. The N-terminal regions are hypothesized to serve as molecular handles during the recruitment of additional viral factors and organization of the viral replication complex [18].

The nsp7–nsp8 complex is responsible for the binding of RNA. It also gives RNA binding capabilities to nsp12 [19]. The activity of nsp12 has also been demonstrated to be regulated by nsp7–nsp8. Nsp7 mutations such as F49A, M52A, L56A, triple mutation of F49A, M52A and L56A, C8G and V11A and nsp8 mutations such as F92A, M90A, M94A have been shown to decrease RdRp activity [10], highlighting the essential roles of these two proteins in the viral replication process. Moreover, nsp7–nsp8 is mostly conserved among different coronaviruses [20] making them potential targets for antiviral drug development owing to their important functions and high conservation.

Several studies have demonstrated that in vitro mutation of viral replication complex proteins in other coronaviruses can sometimes lead to folding defects that affect their function [18] and can sometimes lead to delayed virus growth [21]. These earlier studies collectively underscore the critical importance of nsp7 and nsp8 in viral replication and the impact of other mutations on the viral replication protein complex functionality among coronaviruses, particularly SARS-CoV-2.

Bio-chemoinformatics tools use computational tools that combine bioinformatics and chemoinformatics [22]. This includes techniques in bioinformatics such as sequence assembly and multiple sequence alignment [23] and genomics and proteomics annotation [24], whereas chemoinformatics includes in silico methods such as mutation analysis and protein stability analysis [25,26]. Using these computational tools, researchers can gain insights and pivotal knowledge to understand and explain various biological phenomena on a global scale.

In the present study, we analyzed the global mutation of nsp7 and nsp8 from protein sequence data in May 2022 and April 2023. We further explored the effects of these mutations on viral replication protein complex stability using the computational bio-chemoinformatic analysis. To further investigate the mutational effects of nsp7 and nsp8 on viral replication protein complex stability, critical interaction residues, comparison of bio-chemoinformatic predictions and wet lab experimental results and a computational alanine scan were performed (Supplementary Figure S1). This study is of great importance in understanding the current state of viral protein evolution and how this might affect viral replication mechanisms. Additionally, studying mutations in these proteins can help us gain new insights into identifying amino acid residues for possible targeting and destabilizing the viral replication machinery.

## 2. Materials and Methods

### 2.1. Sequence Mining of Human Isolates and Sequence Alignment

Global protein sequences of SARS-CoV-2 isolated from humans (Homo sapiens) were retrieved from NCBI Beta (https://www.ncbi.nlm.nih.gov/datasets/taxonomy/2697049/) on 27 May 2022 and 17 April 2023. The 2022 dataset had 1,783,299 nsp7 sequences and 1,783,229 nsp8 sequences, and the 2023 dataset had 6,895,947 nsp7 sequences and 6,895,889 nsp8 sequences. The reference sequence for the native nsp7 protein sequence is NCBI Reference Sequence: YP_009725303.1, and the reference sequence for the native nsp8 protein sequence is NCBI Reference Sequence: YP_009725304.1.

The protein sequences were then extracted based on unique sequences and aligned using the default settings of the Geneious alignment method using Geneious Prime software (version 2022.2, Biomatters Ltd., Auckland, New Zealand). Samples identified with unique sequences other than the native protein sequence were referred to as variants. Mutations in the protein sequence were then analyzed further for protein structure analysis.

### 2.2. Conserved Domain Analysis

NCBI Conserved Domain Database (CDD) analysis [27] was performed using the native sequences of nsp7 and nsp8. Nsp7 protein sequence analysis was queried against CDD v3.20–59,693 PSSMs, expected threshold value: 0.010000, composition based on adjustment statistics, and a concise result mode, with a maximum number of hits of 500. The Nsp8 protein sequence analysis was queried against CDD v3.20–59,693 PSSMs, expected threshold value: 0.010000, composition based on adjustment statistics, and a standard result mode, with a maximum number of hits of 500. Critical interaction residues were then generated for nsp7 and nsp8 (Supplementary Dataset S1).

### 2.3. Protein Structural Modeling and Stability Analysis

The RNA-dependent RNA polymerase (RdRp) protein replication complex or viral replication protein complex model containing native sequences of nsp12 (NCBI Reference Sequence: YP_009725307.1), nsp7 and nsp8 (Supplementary Dataset S2) was built using the Robetta server, http://robetta.bakerlab.org (accessed on 1 November 2023), comparative modeling [28] using the reference modeling structure, PDB: 8GWE [29] as a template. The protein sequence query versus PDB template generated a very high sequence identity of 0.98. The comparative model was built from a template structure and aligned using HHSEARCH, SPARKS and Raptor. Loop regions were assembled from fragments and optimized to fit the aligned template structure. The structures generated were superimposed into partial threads before hybrid sampling. The modeled structure was then prepared (e.g., assigning bonds and protonation, fixing structural defects) [9] and refined in ChimeraX [30,31] before protein stability analysis.

Amino acid mutations in nsp7 and nsp8 were then subjected to computational analysis to predict their effects on the stability of the viral replication complex. Alanine scanning was performed by substituting alanine on each amino acid residue. Estimation of Gibbs free energy change values (ΔΔG) were calculated using DDMut, https://biosig.lab.uq.edu.au/ddmut (last accessed on 7 March 2024) which is a deep learning model that captures relationships through its neural network architecture based on published experimental ΔΔG values [32]. The ΔΔG values allow classification of mutation effects as either thermodynamically stabilizing or destabilizing on the protein structure. The ΔΔG change of protein stability is usually defined as follows:

ΔΔG = ΔGmutant − ΔGwild-type

where

ΔGmutant = (G^unfolded^ − G^folded^)

ΔGwild-type = (G^unfolded^ − G^folded^).

In such computational analysis, a ΔΔG < 0 kcal/mol is described as a destabilizing mutation, while a ΔΔG > 0 kcal/mol is a stabilizing mutation.

### 2.4. Data Visualization and Statistical Analysis

Data statistical analysis (e.g., mean, standard error, 95% confidence interval, violin plot) was calculated using DataTab 2024 (e.U. Graz, Austria), https://datatab.net (last accessed on 1 January 2024) [33].

## 3. Results

### 3.1. Global Variation in nsp7 and nsp8 Protein Sequences

The 2022 samples (1,783,299 nsp7 sequences and 1,783,229 nsp8 sequences) generated 506 variants for nsp7 and 1582 variants for nsp8. The 2023 samples (6,895,947 nsp7 sequences and 6,895,889 nsp8 sequences) generated 4537 variants for nsp7 and 14,992 variants for nsp8 (Supplementary Dataset S3).

**Figure 2:**
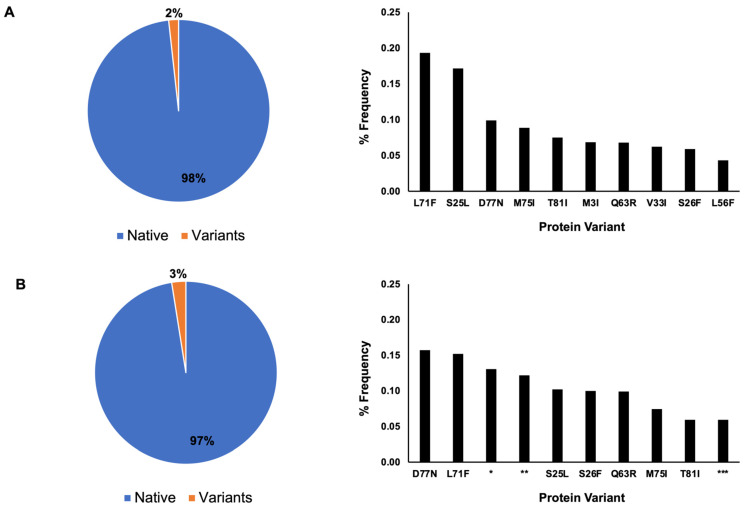
Nsp7 protein sequence distribution. In total, 98% percent of nsp7 contain the native protein sequence, while 2% are variants based on May 2022 data. Single amino acid mutations are dominant among the ten most frequently occurring variants (**A**). Based on April 2023 data (**B**), the native protein is the dominant protein sequence, accounting for 97% of the sequence, with 3% for the variants. Seven single amino acid mutations are the dominant variation. The occurrence of nsp7 with a shorter amino acid sequence (*) and two nsp7 protein sequences containing multiple ambiguous sequences (Xs) (** and ***) are observed in the ten most frequently occurring variants.

The protein sequence distribution for nsp7 showed that 98% of the nsp7 had the native protein sequence and 2% had the variant sequence in 2022, whereas in 2023 (Figure 2), 97% had the native protein sequence and 3% had the variant sequence (Figure 2).

A single amino acid mutation was the dominant type of mutation among the ten most frequently occurring variants for both 2022 and 2023 (Figure 2). In 2022, the frequency of the protein variants was as follows: L71F (0.19%), S25L (0.17%), D77N (0.1%), M75I (0.09%), T81I (0.08%), M3I (0.07%), Q63R (0.07%), V33I (0.06%), S26F (0.06%), and L56F (0.04%). In 2023, the frequency of the protein variants was as follows: D77N (1.43%), L71F (1.05%), shorter amino acid sequence containing several Xs amino acids (denoted as * in Figure 2) (0.56%), protein sequence with multiple ambiguous protein sequence (Xs, denoted as ** in Figure 2) (0.54%), S25L (0.34%), S26F (0.32%), Q63R (0.25%), M75I (0.15%), T81I (0.12%) and another protein sequence with multiple ambiguous protein sequences (Xs, denoted as *** in Figure 2) (0.10%).

**Figure 3:**
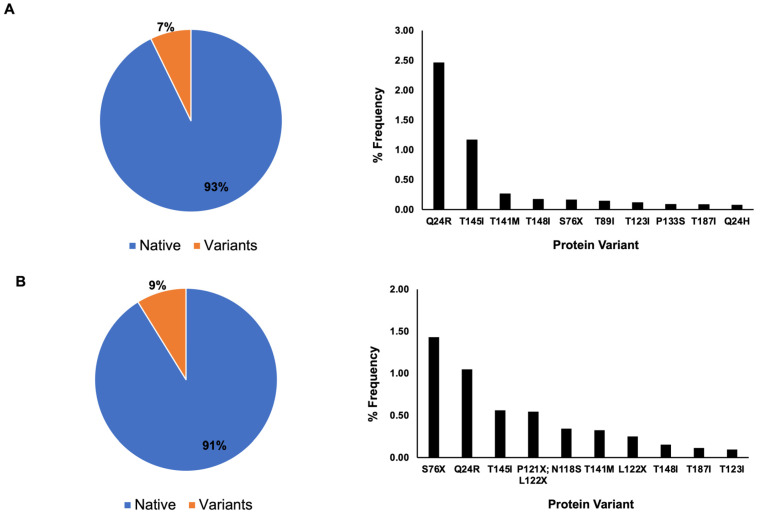
Nsp8 protein sequence distribution. In total, 93% percent of nsp8 contains the native protein sequence, while 7% are variants with mutated sequences based on May 2022 data. Single amino acid mutations are dominant among the ten most frequently occurring variants (**A**). Based on April 2023 data (**B**), the native protein is the dominant protein sequence for nsp8 with 91% occurrence, whereas variants occur at 9%. Single amino acid mutations are the dominant type of mutation, with the exception of a potential double mutation at amino acid positions 121 and 122, where ambiguous amino acid sequences (X) have been reported.

The protein sequence distribution for nsp8 showed that 93% of the nsp8 sequences have the native protein sequence and the remaining 7% as variants in 2022 (Figure 3), whereas in 2023, 91% have the native protein sequence and 9% have the variant sequence (Figure 3).

Similar to the nsp7 protein sequence analysis, a single amino acid mutation was the dominant mutation among the ten most frequently occurring protein sequence variants for both 2022 and 2023 for nsp8 (Figure 3). In 2022, the frequency of variants was as follows: Q24R (2.46%), T145I (1.17%), T141M (0.26%), T148I (0.17%), S76X (0.16%), T89I (0.14%), T123I (0.12%), P133S (0.09%), T187I (0.08%) and Q24H (0.07%). In 2023, the frequency of protein variants was as follows: S76X (1.43%), Q24R (1.05%), T145I (0.56%), P121X and L122X (0.54%), N118S (0.34%), T141M (0.32%), L122X (0.25%), T148I (0.15%), T187I (0.12%) and T123I (0.10%). The mutation of the amino acid threonine into isoleucine was the most frequently occurring amino acid mutation for both 2022 and 2023 (Figure 3).

### 3.2. Predicted Protein Stability of the Most Frequently Occurring Protein Variants

To determine the effects of the mutations on the viral replication protein complex, calculation of the ΔΔG of the mutant protein was performed. The effects of nsp7 mutation on the C-chain of the viral replication complex were modeled (Table 1). Among the most frequently occurring nsp7 mutations, seven were found to cause destabilizing effects, whereas three were found to cause stabilizing effects. L56F and L71F had the greatest destabilizing effect (−1.16 kcal/mol; −1.13 kcal/mol), whereas S26F and M75I (0.39 and 0.35 kcal/mol) had the greatest stabilizing effect on the viral replication complex.

For the effects of nsp8, we explored the effects of the mutation on each of the B and D chains and its overall effect (B, D mutation) (Table 2). Among the most frequently occurring single amino acid nsp8 mutations (excluding those containing ambiguous amino acid), nine mutations were found to cause overall stabilizing effects on the viral replication complex, with one mutation (P133S) causing destabilizing effects (−3.34 kcal/mol).

**Figure 4:**
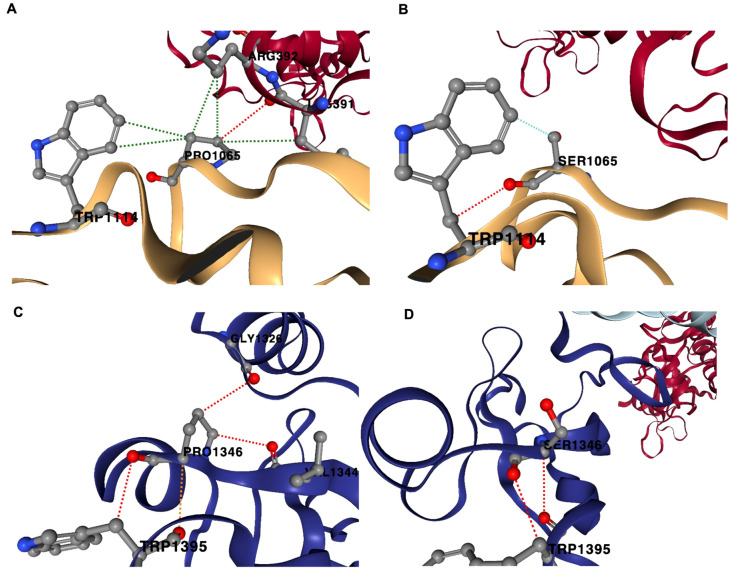
Non-covalent interactions in P133S variant. The native protein Pro-133 (PRO1065; B-chain) forms H-bond with Lys-391, A-chain (nsp12); hydrophobic interactions with Trp-182 (TRP1114; B-chain), Arg-392 (A-chain) and Lys-391 (A-chain) (**A**). In the variant sequence, Ser-133 (SER1065; B chain) forms H-bond with Trp-182 (TRP1114; B-chain); van der Waals interaction with Trp-182 (TRP1114; B-chain) (**B**). Pro-133 (PRO1346; D-chain) forms H-bonds with Gly-113 (GLY1346; D-chain), Trp-182 (TRP1395; D-chain) and Val-131 (VAL1344; D-chain) (**C**). Ser-133 (D-chain) forms one H-bond with Trp-182 (TRP1395, D-chain) (**D**). Red-dotted lines represent H-bonds, green-dotted lines represent hydrophobic interactions and cyan-dotted lines represent van der Waals interaction.

In the case of the P133S variant, the native sequence Pro-133 amino acid residue stabilized the structure by forming several inter-chain and intra-chain interactions (Figure 4). Pro-133 (B-chain) forms an inter-chain H-bond with Lys-391 of the A-chain (nsp12). Several hydrophobic interactions occur between Pro-133 (B-chain) and residues such as Trp-182 (B-chain), Arg-392 (A-chain) and Lys-391 (A-chain). However, in the variant sequence, Ser-133 forms only one intrachain H-bond with Trp-182 (B-chain). Ser-133 also does not form hydrophobic interactions like that of P-133; rather, Ser-133 forms a weaker van der Waals interaction with Trp-182 (B-chain) and Ser-133 (B-chain). In the D-chain, Pro-133 forms three intra-chain H-bonds with residues Gly-113, Trp-182 and Val-131. However, the variant Ser-133 (D-chain) only forms one H-bond with the residue Trp-182. Pro-133 had more stability than Ser-133 due to the multiple H-bonding and multiple hydrophobic interactions it formed with its neighboring atomic environment compared with the Ser-133 variant.

A single amino acid substitution of threonine to isoleucine is a common mutation in nsp8. Analysis showed that these mutations stabilize the viral replication protein complex. We observed that during this mutation, an increased number of non-covalent interactions, in particular hydrophobic interactions, occurred. In T145I, the native sequence Thr-145 can form a polar interaction with Ile-156 in the B-chain. With a mutation to Ile-145, two polar interactions occur between Ile-145 and Asp-143. In the case of the T148I variant, additional hydrophobic interactions occur between the variant, Ile-148, and Leu-153 in the D-chain. In Thr-148 (D-chain), no hydrophobic interactions were observed, whereas the mutant Ile-148 formed a hydrophobic interaction with Leu-76 of nsp7 (C-chain). In T123I (B-chain), Thr-123 formed only one hydrophobic interaction with Ile-270 (A-chain, nsp12). Upon mutation to Ile-123, inter- and intra-chain hydrophobic interactions occur with Leu-270 (A-chain), Ile-119 (B-chain) and Ile-106 (B-chain). In the T187I (B-chain), Thr-187 and Lys-127 form hydrophobic interactions. The variant Ile-187 forms hydrophobic interactions with Lys-127, Met-137 and Ile-185 (B-chain) (Supplementary Dataset S4).

Because other frequently occurring mutations such as S76X and L122X contained ambiguous amino acid sequences, we simulated all possible 19 amino acid mutations that might occur for the variants. Among the possible mutations at the 76th amino acid residue position of nsp8, an S76P mutation would render the most destabilizing effect (−1.39 kcal/mol), whereas an S76Y mutation would cause a stabilizing effect (2.27 kcal/mol) (Table 3). At the 122nd position of nsp8, an L122G mutation would render the most destabilizing effect (−2.42 kcal/mol), whereas an L122W mutation (0.95 kcal/mol) would cause a stabilizing effect (Table 4).

One of the most frequently occurring potential variants of nsp8 is a potential double mutation at the two amino acid positions, 121st and 122nd. To predict the potential effect of double amino acid substitutions on these sites, we performed various possible amino acid substitutions via permutations with repetition (Supplementary Dataset S5). Table 5 shows that a double substitution to glycine (P121G,L122G) causes the greatest destabilization (−4.04 kcal/mol), followed by P121D,L122G; P121T,L122G; and P121S,L122G (−3.98, −3.91 and −3.82 kcal/mol).

On the other hand, a mutation with only on the 121st amino acid position from a proline (P) to a glutamic acid, E (P121E), will cause the greatest stabilizing effect (1.65 kcal/mol). This is followed by a mutation to a Q (P21Q) (1.52 kcal/mol) and a double mutation of P121E and L122F (1.19 kcal/mol).

### 3.3. Mutation Effects on Critical Amino Acid Positions of nsp7 and nsp8

To further explore amino acid residues that might be critical in nsp7 and nsp8 for protein interactions, a conserved domain database (CDD) analysis was performed. CDD analysis allows identification and characterization of amino acid residues within a protein sequence that are structurally and evolutionarily conserved across different virus species. Protein homologues across different species of related viruses were used for protein alignment (Supplementary Figure S2).

The alignment used 27 nsp7 protein sequences and its homolog across different species, whereas the alignment for nsp8 comprised 30 protein sequences across different species. CDD analysis revealed conservation of three amino acid residues for nsp7 and nsp8. These critical interaction residues for nsp7 are Lys-7, His-36 and Asn-37, and the critical interaction residues for nsp8 are Lys-58, Pro-183 and Arg-190.

**Figure 5:**
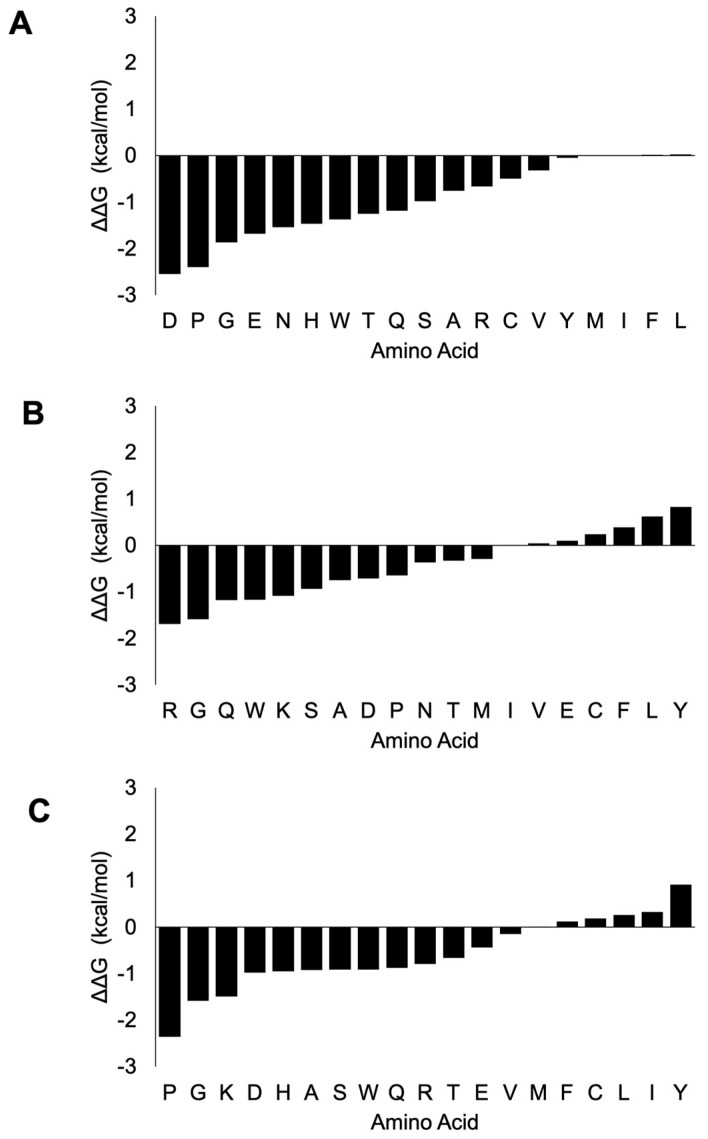
ΔΔG change upon mutation of the three critical interaction residues in nsp7. The mutation of Lys-7 shows a destabilizing effect with only I, F and L amino acids showing minimal stabilizing effects (**A**). The mutation of His-36 has mostly destabilizing effects, with only F, C, L, I and Y mutations having stabilizing effects (**B**). Similarly, mutation at Asn-37 causes destabilizing effects except Y, I, L, C, F and M amino acid mutations, which render stabilizing effects (**C**). The x-axis shows the ΔΔG (kcal/mol) and the y-axis shows the amino acid substitution.

To further understand the potential stability changes upon mutation of these critical interaction residues, we examined the changes in the ΔΔG energy upon substitution to any of the remaining 19 amino acids. In the case of nsp7 (Figure 5, Supplementary Dataset S6), the mutation of Lys (K) at the 7th amino acid position to D (−2.55 kcal/mol), P (−2.4 kcal/mol) and G (−1.87 kcal/mol) amino acids would cause the most destabilizing effect, whereas the mutation to any of the three amino acids: L (0.03 kcal/mol), F (0.02 kcal/mol) and I (0.01 kcal/mol) would cause a slightly stabilizing effect, with a mutation to M (0.00 kcal/mol) causing a neutral mutation. The mutation at the 36th position from amino acid H to R (−1.69 kcal/mol), G (−1.59 kcal/mol) and Q (−1.18 kcal/mol) would cause the greatest destabilizing effects. Mutation to any of the seven amino acids, namely Y (0.83 kcal/mol), L (0.62 kcal/mol), F (0.39 kcal/mol), C (0.24 kcal/mol), E (0.1 kcal/mol), V (0.04 kcal/mol) and I (0.01 kcal/mol), would cause stabilizing effects. The mutation at the 37th position from amino acid N to P (−2.36 kcal/mol), G (−1.59 kcal/mol) and K (−1.49 kcal/mol) would cause the most destabilizing effects, whereas mutation to any of the six amino acids Y (0.91 kcal/mol), I (0.33 kcal/mol), L (0.26 kcal/mol), C (0.19 kcal/mol), F (0.12 kcal/mol) and M (0.01 kcal/mol) could cause stabilizing effects. Our analysis showed that thermodynamically, most amino acid substitutions of these three critical interaction residues render destabilizing effects.

**Figure 6:**
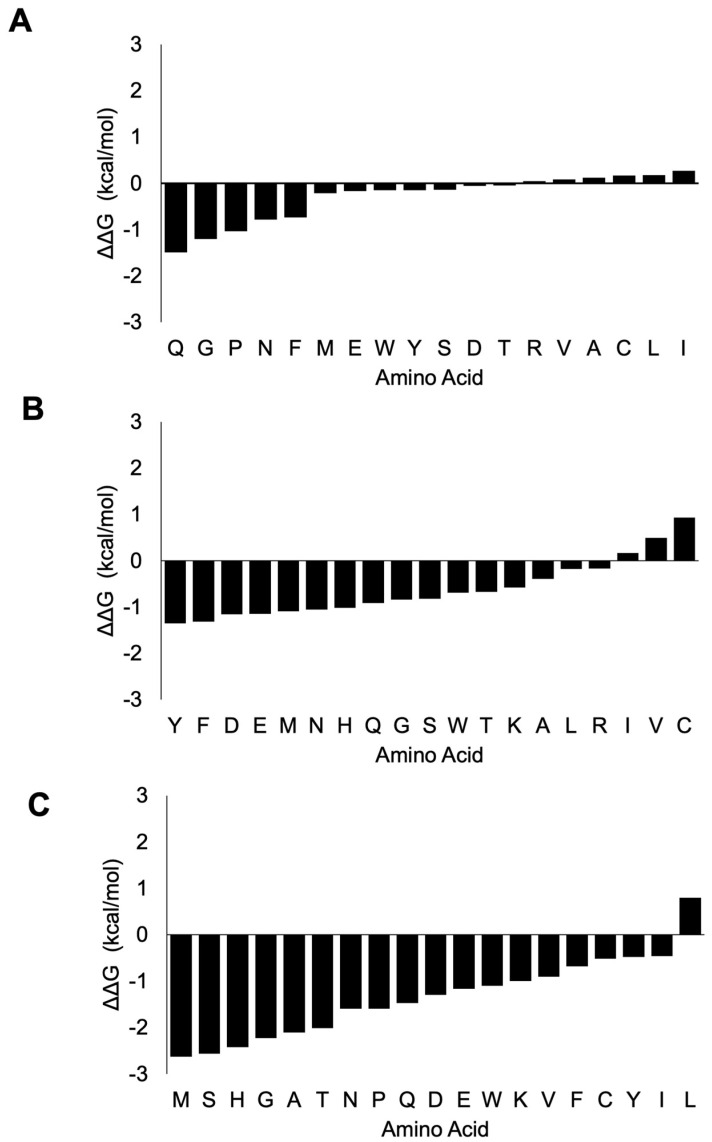
ΔΔG change upon mutation of the three critical interaction residues in nsp8. The mutation of Lys-58 shows a destabilizing effect with I, L, C, A, V and R amino acids showing stabilizing effects (**A**). The mutation of Pro-183 has mostly destabilizing effects, with only C, V and I amino acid mutations causing stabilizing effects (**B**). Mutation at Arg-190 has largely destabilizing effects, with the exception that only L amino acid has stabilizing effects (**C**). The x-axis shows the ΔΔG (kcal/mol) and the y-axis shows the amino acid substitution.

In the case of nsp8 (Figure 6, Supplementary Dataset S7), mutation of Lys-58 would render a mostly destabilizing effect especially with amino acids H (−1.55 kcal/mol), Q (−1.49 kcal/mol) and G (−1.2 kcal/mol). Stabilizing effects were rendered with mutations to I (0.27 kcal/mol), L (0.18 kcal/mol), C (0.17 kcal/mol), A (0.12 kcal/mol), V (0.08 kcal/mol) and R (0.05 kcal/mol).

A mutation at Pro-183 (P) would mostly cause a destabilizing effect, especially with the mutations to Y (−1.35 kcal/mol), F (−1.32 kcal/mol), D (−1.16 kcal/mol), E (−1.15 kcal/mol) and M (−1.09 kcal/mol). On the other hand, stabilizing effects were observed with mutations to C (0.93 kcal/mol), V (0.49 kcal/mol) and I (0.17 kcal/mol). Mutation to any other amino acid except L amino acid (0.80 kcal/mol) of Arg-190 would have a destabilizing effect on the viral replication protein complex.

### 3.4. Comparison of Bio-Chemoinformatic Calculations and Predictions with Wet Lab Experimental Results

To confirm the reliability of bio-chemoinformatic calculations and predictions and to gain understanding of their biological significance, we simulated known mutations of nsp7 and nsp8 based on wet lab experiments reported by Biswal, 2021 [10].

Reported mutations of nsp7 which have shown to decrease the RdRp efficiency include F49A, M52A, L56A, triple mutation of F49A, M52A, L56A, C8G and V11A. In the case of F49A, M52A and L56A, experimental evidence has shown that a triple mutation of F49A, M52A and L56A disrupted RdRp efficiency greatly compared to the individual mutation components.

Table 6 shows that the destabilizing mutations for nsp7 based on the wet lab experimental results are in agreement with our bio-chemoinformatics analysis. A triple mutation of F49A, M52A and L56A (−3.46 kcal/mol) was found to be higher than the individual mutation effects: F49A (−2.99 kcal/mol), M52A (−2.12 kcal/mol) and L56A (−3.09 kcal/mol).

Mutation of nsp7 N37V was reported to have no detrimental effect to the nsp7–nsp8 complex but caused decrease in RdRp activity when it was part of the viral replication protein complex. In this regard, we modeled three situations: (1) mutation of nsp7 N37V in nsp7–nsp8 dimer complex (PDB: 6YHU), (2) mutation of nsp7 N37V in the nsp7–nsp8 heterotetrameric complex using the X-ray crystal structure of the wet lab experiments (PDB: 7JLT) and mutation of nsp7 N37V as part of the viral replication protein complex.

Bio-chemoinformatic analysis showed that N37V has no detrimental effect (stabilizing or neutral effect) on both the nsp7–nsp8 dimer complex (0.13 kcal/mol) and nsp7–nsp8 heterotetramer complex (0.22 kcal/mol) but has a destabilizing effect (−0.15 kcal/mol) or reduction on the RdRp efficiency when introduced in the viral replication complex.

To further confirm biological significance of the bio-chemoinformatic calculations and predictions (Table 7), we simulated the nsp8 mutations based on reported wet lab experiments. Experimental evidence has shown that F92A, M90A and M94A have destabilizing effects on the RdRp efficiency [10].

Table 7 shows that our biochemoinformatic analyses are in agreement with the observed experimental results in which destabilizing effects were observed. The destabilizing effects were: F92A (−3.06 kcal/mol), M90A (−1.39 kcal/mol) and M94A (−1.94 kcal/mol).

Overall, our analysis has shown that bio-chemoinformatic analyses are in good agreement with the wet lab experimental results. Moreover, bio-chemoinformatic results which are stabilizing render neutral or no detrimental effect or possibly improve efficiency to some extent to the RdRp, whereas destabilizing effects render a decrease in RdRp efficiency.

### 3.5. Individual Amino Acid Residue Contributions to Protein Complex Stability

To further explore the contributions of each amino acid residue to the stability of the viral replication complex, we conducted an alanine scan on each of the protein chains. Each of the non-alanine amino acid residues was mutated to alanine, and our analysis showed that most of the amino acid residue sites of the viral replication complex are prone to destabilization or are thermodynamic hotspots, whereas some portions are neutral or stabilizing sites. In total, 84.1% of the nsp12 protein, 76.5% of the nsp8: B-chain, 82.4% of the nsp8: D-chain, and 80.8% of the C-chain are prone to destabilization upon alanine mutation. A simultaneous alanine scan of nsp8 at both the B-chain and D-chain revealed that 48% of the total amino acid residues were prone to destabilization (Supplementary Dataset S8).

On the other hand, the percentage of amino acid residues that would render stabilization upon alanine mutation are: 15.9% of nsp12 (A-chain), 23.5% of nsp8 (B-chain), 17.7% of nsp8 (D-chain) and 19.2% of nsp7 (C-chain). Simultaneous alanine scans at both the B-chain and D-chain showed that 42% of the total amino acid residues were neutral sites (Supplementary Dataset S8).

**Figure 7:**
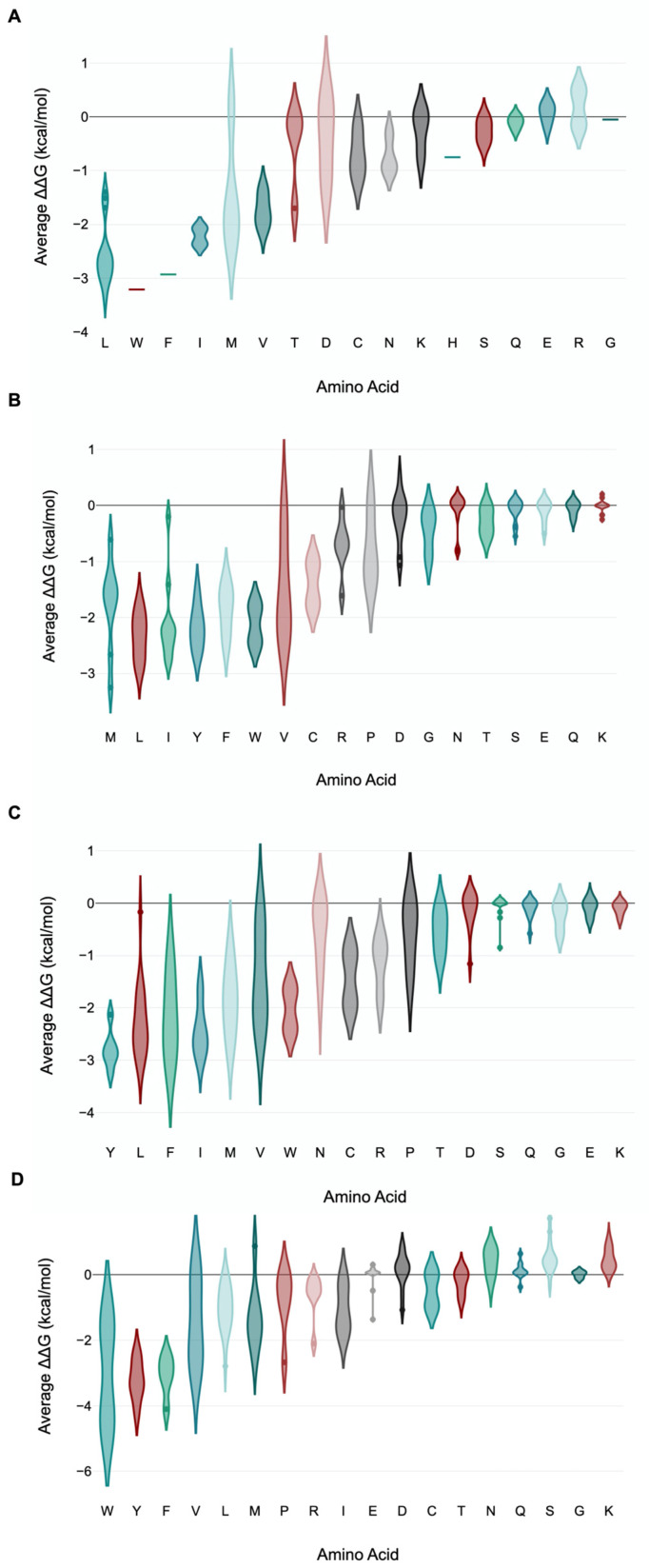
Violin plot of amino acid residue contributions to viral replication complex stability. The violin plot shows the distribution of the different destabilizing/stabilizing effects of each amino acid residue when substituted with alanine. Mutation of some amino acid residues to alanine is found to have greater destabilizing effects than other amino acids. These amino acids that render greater stability to the viral replication complex include W, F, L and I in nsp-7 (**A**); L, Y, F, W and I in nsp8 (B-chain) (**B**); Y, I, L, F and W in nsp8 (D-chain) (**C**); and Y, F and W in nsp8 (combined B- and D-chain) (**D**). The x-axis shows the amino acid, whereas the y-axis shows the average ΔΔG for the amino acids analyzed.

**Figure 8:**
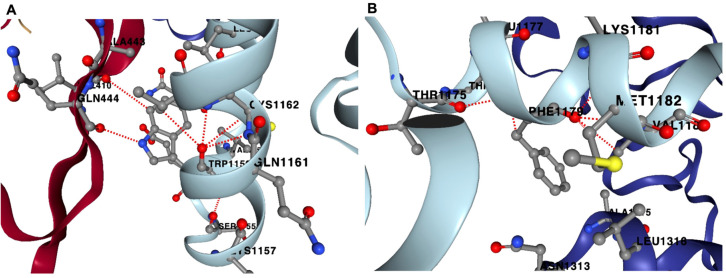
Hydrogen bonding sites for Trp-29 and Phe-49 in nsp7. Nsp7 has only one tryptophan, Trp-29, which can form H-bonds with Gln-444 and Val-410 of the A-chain (nsp12) (**A**). Nsp7’s only phenylalanine, Phe-49, can form multiple H-bonds within the C-chain, which includes Met-52 (MET1182; C-chain), Thr-45 (THR1175; C-chain) and Thr-46 (THR1176; C-chain) (**B**). Red-dotted lines represent H-bonds.

We further examined the overall contribution of the amino acids of nsp7 and nsp8 to the overall stability of the protein complex. In the case of nsp7 (C-chain), the amino acids W (−3.21 kcal/mol), F (−2.93 kcal/mol), L (−2.56 ± 0.58 kcal/mol) and I (−2.22 ± 0.21 kcal/mol) demonstrated the greatest destabilizing effect (<−2.0 kcal/mol average) during alanine substitution (Figure 7A; Supplementary Dataset S9). Nsp7 has only one tryptophan and one phenylalanine, Trp-29 and Phe-49. Trp-29 contributes to the stability of the protein complex through interchain interactions between the A-chain and C-chains. An important interaction is H-bonding with Gln-444 of nsp12 (A-chain) and Val-410 of nsp12 (A-chain). Furthermore, Phe-49 of nsp7 forms several H-bonds with neighboring amino acids in the C-chain, which helps stabilize the complex. H-bonding occurs between Phe-49 and amino acid residues such as Met-52, Val-53, Thr-45 and Thr-46 within the C-chain (Figure 8).

Examining nsp8 (B-chain), the amino acids L (−2.33 ± 0.41 kcal/mol), Y (−2.18 ± 0.39 kcal/mol), W (−2.12 ± 0.45 kcal/mol) and I (−2.08 ± 0.76 kcal/mol) exhibited the greatest destabilizing effect when substituted with alanine (Figure 7B; Supplementary Dataset S9). An alanine scan of nsp8 at the D-chain showed the greatest average destabilizing effect when the amino acids Y (−2.79 ± 0.38 kcal/mol), I (−2.43 ± 0.5 kcal/mol), L (−2.26 ± 0.7 kcal/mol), F (−2.16 ± 0.83 kcal/mol) and W (−2.03 ± 0.54 kcal/mol) were substituted with alanine (Figure 7C).

Simultaneous alanine scan of nsp8 at both the B-chain and D-chain showed Y (−3.21 ± 0.63 kcal/mol), F (−3.08 ± 0.64 kcal/mol) and W (−3.01 ± 2.05 kcal/mol) with the greatest average destabilizing effect (Figure 7D). The average value for both chains was higher than the average values for each of the individual chains. Moreover, average values for the mutational effect for both chains showed a stabilizing effect for amino acids such as G (0 ± 0.11 kcal/mol), Q (0.12 ± 0.25 kcal/mol), D (0.13 ± 0.52 kcal/mol), N (0.39 ± 0.43 kcal/mol), K (0.47 ± 0.34 kcal/mol) and S (0.52 ± 0.5 kcal/mol). These positive average stabilizing effect values were not observed for these amino acids for each of the individual B and D chains (Supplementary Dataset S9).

Three amino acid residues of nsp8 showed the greatest destabilizing effect (<−4.0 kcal/mol) in both the B-chain and D-chain. These are Trp-154 (−4.46 kcal/mol), Tyr-135 (−4.17 kcal/mol) and Phe-15 (−4.1 kcal/mol) (Supplementary Dataset S9).

Destabilizing effects were mostly caused by the disruption of the H-bonding that forms in their respective atomic environments. Trp-154 forms four intrachain H-bonds with Phe-147, Leu-189, Tyr-149 and Ala-126. In the D-chain, it forms two H-bonds with Phe-147 and Tyr-149. In Tyr-135, it forms three H-bonds in the B-chain, namely with Lys-139, Tyr-138 and Ile-172, while it forms two H-bonds in the D-chain, with Tyr-138 and Lys-139. Aside from several intra-chain H-bonds at the B-chain with amino acid residues Ala-18, Gln-19, Ser-11 and Tyr-12, the native amino acid residue Phe-15 forms an aromatic interaction with a neighboring Phe-49. The same aromatic interaction also occurs at the D-chain of the viral complex with Phe-49. Phe-15 also forms two H-bonds with Tyr-12 and Ser-11 (Supplementary Dataset S10).

## 4. Discussion

A global study of mutations in viral replication is important to understand viral evolution and drug resistance [34], disease pathogenesis [35] and the development of antiviral strategies [36]. These studies provide insights and knowledge into the molecular mechanisms underlying viral replication at the population level and offer a foundation for the development of targeted therapeutic interventions and the design of novel antiviral agents [36,37].

In this regard, we analyzed the nsp7 and nsp8 protein sequences available from 2022 to 2023 at NCBI. Our analysis of global mutations for 2022 and 2023 showed that more than 90% of the global protein sequences conserve the native protein sequences. A prior study in 2021–2022 also reported similar findings on the percentage of native protein sequences for nsp7 and nsp8 [38], although the study did not further investigate the effect of these mutations on the viral replication protein complex.

In 2021, only S25L (1.70%) and S26F (0.28%) have percentage frequencies of occurrence greater than 0.10% for nsp7. The remaining mutations were at 0.01–0.02%. For nsp8, only M129I (0.35%) and I156V (0.33%) have frequencies greater than 0.10%, with the remaining variants in the frequency range of 0.01–0.06% [9]. Our recent data for 2022 and 2023 show that the percentage frequency distribution of variants for nsp7 did not exceed 0.20% (Figure 2). S25L and S26F, along with D77N and L71F, are the most frequently occurring variants of nsp7 for 2022 and 2023. For nsp8, M129I and I156V are not in the 10 most frequently occurring variants for 2022–2023. Meanwhile, S76X and Q24R are the two most frequently occurring variants, with percentage frequencies greater than 1% for 2023. Out of the ten most frequently occurring variants (Table 2), only the P133S mutation rendered a stabilizing effect, whereas the remaining variants rendered a stabilizing effect. We also simulated possible mutations for the most frequently occurring mutations in nsp8, which contains one and two ambiguous amino acid sequences. Ambiguous amino acid sequences often arise due to low quality or poor sequencing data [39], degenerate genetic codes in which multiple codons may code for the same amino acid [40,41], and genetic variations such as insertions, deletions or mutations [42,43]. In S76X, mutation to P, G, N and D amino acids would have destabilizing effects, whereas any other amino acids would have neutral or stabilizing effects. In L122X, a mutation to glycine causes the greatest destabilizing effect. The same effect of glycine substitution was observed in the two amino acid substitutions, P121X and L122X. In the P121X, L122X variant, mutation of the 122nd amino acid to G amino acid with Pro-121 mutating to G, D, T, S and N amino acids would cause the greatest destabilization, while stabilizing effects occurred when there was no mutation on the 122nd position and a mutation to E and Q amino acids occurred at the 121st amino acid position. For the double mutations of P121X and L122X, DDMut has been tested for high accuracy for three simultaneous mutations. It is recommended as a future study for P121X, L122X to be compared with other in silico analyses for four simultaneous mutations. In summary, most of the frequently occurring mutations for nsp7 are predicted to cause a destabilizing effect, whereas mutations for nsp8 would render a stabilizing effect on the viral replication protein complex.

Mutation of threonine to isoleucine at different positions was notable in the most frequently occurring variants of nsp8 (Table 2). Our analysis showed that mutations from threonine to isoleucine would have an overall stabilizing effect on the viral replication complex. The substitution of threonine with isoleucine can often influence protein stability changes through hydrophobic interactions, hydrogen bonding and side chain packing [44,45]. Hydrophobic isoleucine can enhance hydrophobic interactions within the protein core, which contribute to stability [44]. This stability causes increased thermal stability and hydrophobicity through improved internal packing and increased hydrophobic interactions [46]. The role of hydrophobic interactions was noted in our analysis of nsp8, in which an increased number of hydrophobic interactions with the substitution of threonine and isoleucine was observed for the mutants. In proteins, such as the villin headpiece subdomain, conformation is mainly stabilized through hydrophobic interactions [47].

In the context of viral protein mutations, threonine to isoleucine mutations have been associated with functional changes, altering viral infectivity and interactions with host cellular processes. A threonine to isoleucine mutation has also been reported in different proteins, such as the polymerase protein of murine leukemia viruses [48], capsid of RNA viruses [49] and the P7 protein of hepatitis C virus [50]. In terms of functionality, a threonine to isoleucine mutation at position 544 of the spike glycoprotein of Zaire ebolavirus has been frequently observed in past outbreaks and has been shown to have a potential role in infection efficiency [51]. In human immunodeficiency virus type-1 (HIV-1), a T24I mutation of the nucleocapsid protein has been reported as a second-site suppressor that causes the rescue of replication and RNA packaging [52]. Hence, thermodynamically, a threonine to isoleucine mutation can cause protein stability and can cause favorable biological effects on the virus, such as an increase in infection rate and replication rescue.

We further analyzed mutational effects on the critical interaction residues that were identified using conserved domain database analysis. Our analysis revealed that the critical interaction residues for nsp7 are Lys-7, His-36 and Asn-37. These three amino acids are incongruent with experimental studies proposing the potential critical role of these three amino acids in the potential interaction of the nsp7/nsp8/nsp12 polymerase complex with RNA [21]. For nsp8, the three potential critical interaction residues are Lys-58, Pro-183 and Arg-190. These three amino acids are also incongruent with some studies that have proposed their potential critical roles, with Pro-183 and Arg-190 postulated to be involved in nsp12 binding and Lys-58 might be critical for Nsp8–RNA interactions [21,53].

Our findings showed that most amino acid substitutions on these sites for nsp7 and nsp8 would render an overall destabilizing effect on the viral replication complex. This was quite evident, in particular with the mutation of Lys-7 of nsp7 in which substitution of any other amino acid would mostly cause destabilization. In the case of Arg-190 of nsp8, mutation to any other amino acid except for L amino acid will cause destabilization. In our 2023 global analysis, we noted that certain mutations at these critical amino residues have been sequenced. For Lys-7 of nsp7, K7R (*n* = 444) is the most frequently occurring variant in the dataset, followed by K7Q (*n* = 14) and K7N (*n* = 6). For His-36 and Asn-37, some mutations were observed but at a low frequency. These include H36T (*n* = 4), H36P (*n* = 2), H36Q (*n* = 2), N37S (*n* = 6), N37N (*n* = 6), N37D (*n* = 2) and N37K (*n* = 2). For nsp8, no mutation so far has been sequenced for Lys-58, whereas variants for Pro-183 and Arg-190 have been sequenced at low frequencies. These variants are P183S (*n* = 10), P128L (*n* = 4), R190A (*n* = 4), R190P (*n* = 8) and R190P (*n* = 2). Based on our computational analysis, we predict that these mutations might cause destabilizing effects on the viral replication complex. Overall, our computational thermodynamic data are in agreement with an earlier hypothesis that these three respective amino acid residues of nsp7 and nsp8 are critical interaction residues conserved across different non-human viral isolates. Disrupting these amino acid sites may be further explored for further studies as potential target sites.

We also simulated wet lab experiments using bio-chemoinformatic calculations to confirm the reliability of our methods and to gain biological significance. Our results showed good agreement with previously reported effects of mutations of nsp7 and nsp8 on the viral replication protein complex [10]. The nsp7 triple mutation of F49A, M52A and L56A demonstrated the greatest destabilizing effect compared to their individual mutations (Table 6). This was consistent with the observed wet lab experiments where the triple mutation caused a greater decrease in RdRp efficiency. Our results also showed destabilizing effects with other mutations such as C8G and V11A, which were also reported to decrease the RdRp efficiency. Moreover, our analysis showed that the nsp7 N37V mutation caused stabilizing or neutral effects when expressed as part of the nsp7–nsp8 dimer and nsp7–nsp8 heterotetramer complex. Destabilizing effects were predicted when it is expressed as part of the viral replication protein complex or the nsp12–nsp7–nsp8 supercomplex. These results were in agreement with the wet lab experiments, which reported no detrimental effect to the nsp7–nsp8 complex but notably decreased RdRp activity when expressed as part of the viral replication protein complex. Furthermore, mutations of nsp8 such as F92A, M90A and M94A, which we predicted to be destabilizing, have been shown in the wet lab experiments to have decreased RdRp efficiency. This suggests that our bio-chemoinformatics results showing stabilizing or neutral effects render no detrimental effect or possibly increased activity to some extent to the RdRp efficiency, while destabilizing effects render decreased RdRp efficiency. Reduced RdRp activity has been shown to substantially slow down viral replication in RNA viruses such as in tick-borne flavivirus [54] and can alter the RNA synthesis process in tomato mosaic virus [55]. Also, inhibitors of RdRp of SARS-CoV-2 such as remdesivir slow down viral replication by reducing and inhibiting the viral RdRp efficiency [56]. This highlights the biological significance of the stabilizing and destabilizing effects of mutations on the viral replication protein complex in the context of viral replication fitness.

Another aspect that we examined in this study is the alanine scan of the amino acid residues comprising the viral replication complex. Our analysis has shown that most regions of the viral replication complex are potential hotspot residues or thermodynamically destabilizing sites, whereas a few are neutral or stabilizing sites. Simultaneous alanine scans of the B-chain and D-chain showed that 48% of the amino acid residues were potential hotspots and 42% were neutral sites. In contrast, individual alanine scans of the B-chain and D-chain showed that 82.4% were potential hotspots for the B-chain and 80.8% were potential hotspots for the D-chain. The difference in the number of potential hotspots when both the B- and D-chains are present can be attributed to possible interchain and intrachain interactions within the protein complex [57], allosteric effects [58] and conformational changes [59,60]. The destabilizing effect during alanine mutations in individual chains often arises from the disturbance of critical interactions within each chain, which lead to decreased stability. When both chains are mutated simultaneously to alanine, it can often lead to the formation of favorable interactions at the interface between the chains, resulting in a stabilizing effect on the overall complex [61]. In the case of proteins such as nsp8, which form two chains in a complex, a simultaneous poly-alanine scan would be a better technique to determine the effect of each amino acid residue on overall protein stability.

Amino acids such as leucine, tryptophan, phenylalanine and isoleucine in nsp7 and tryptophan, tyrosine and phenylalanine in nsp8 are prone to destabilization when substituted with alanine. Our results agree with those of a previous study that used energy per residue decomposition to predict amino acid hotspots in which tyrosine, phenylalanine and leucine were some predicted hotspot candidates [11]. Hotspot amino acid residues have been found to be enriched in forming H-bonds [11,62], such as in the case of Tryp-29 and Phe-49 of nsp7 and Trp-154, Tyr-135 and Phe-15 of nsp8 in our analysis. These amino acid residues exhibited the greatest destabilizing effect owing to the disruption of hydrogen bonds that they, respectively, form within the viral replication protein complex. Exploration of these residues as potential hotspot residues as target candidates can be further performed for confirmation.

The present study has studied extensively the temporal mutation frequencies of nsp7 and nsp8, identified critical interaction residues, confirmed previously reported wet lab results and identified new amino acid residue targets for possible drug development. In this aspect, as we utilized the native sequence of nsp12 in our models, a possible mutation of nsp12 in combination with mutations of nsp7 and nsp8 can be performed to study multi-chain mutations as a future direction. With data on mutations of spike proteins being richly available in the literature [63,64], transmission and spread models based on mutations of infectivity-related protein and replication-related proteins of SARS-CoV-2 [65,66,67] can be of great interest to assimilate relevant data in tracking the molecular evolution, distribution and implications on the global epidemiological trend of the virus. This would allow the development of robust methods to mitigate the spread of the virus and to develop high-efficacy and high-specificity drugs.

## 5. Conclusions

The present study analyzed the global mutation of nsp7 and nsp8 in 2022 and 2023, in which certain mutations have significant effects on the stability of the viral replication complex. Most of the frequently occurring mutations in nsp7 were predicted to destabilize, whereas mutations in nsp8 were predicted to cause stabilization. The substitution of threonine with isoleucine in nsp8 was found to occur frequently in the global population. This mutation can lead to increased stability and may cause potential functional changes. Moreover, critical interaction residues for nsp7 and nsp8 have been identified, and the effects of mutations on these sites caused destabilization. Bio-chemoinformatic predictions were in good agreement with previously reported wet lab experimental results. Furthermore, potential hotspot residues for nsp7 and nsp8 have been predicted with amino acids such as tryptophan, phenylalanine and tyrosine, proposing their possible role as amino acid residues for targeting. The present study provided an intensive study of the mutations of nsp7 and nsp8 and their effects on the stability of the viral replication protein complex. This has allowed a better understanding of the current state of viral protein evolution, the possible effect on viral replication mechanisms and insights into new possible protein target sites.

## Figures and Tables

**Table 1:** Most frequently occurring mutations in nsp7 and their mutational effects based on ΔΔG values.

Mutation	Chain	ΔΔGStability Prediction (kcal/mol)	Effect
L56F	C	−1.16	Destabilizing
L71F	C	−1.13	Destabilizing
S25L	C	−0.68	Destabilizing
M3I	C	−0.54	Destabilizing
D77N	C	−0.38	Destabilizing
V33I	C	−0.24	Destabilizing
T81I	C	−0.01	Destabilizing
Q63R	C	0.05	Stabilizing
M75I	C	0.35	Stabilizing
S26F	C	0.39	Stabilizing

**Table 2:** Most frequently occurring mutations in nsp8 and their mutational effects based on ΔΔG values.

Mutation	Chain	Individual ΔΔGStability Prediction (kcal/mol) B; D Chains	ΔΔG Over-All Stability Prediction (kcal/mol)	Effect
P133S	B, D	−1.22; −0.62	−3.34	Destabilizing
Q24H	B, D	−0.02; −0.03	0.17	Stabilizing
T89I	B, D	0.07; 0.11	0.89	Stabilizing
T141M	B, D	−0.13; 0.6	1.02	Stabilizing
T145I	B, D	0.07; 0.08	1.18	Stabilizing
Q24R	B, D	−0.04; −0.12	1.19	Stabilizing
T123I	B, D	0.05; 0.0	1.27	Stabilizing
N118S	B, D	−0.8; −0.46	1.96	Stabilizing
T148I	B, D	0.18; 0.14	2.23	Stabilizing
T187I	B, D	0.54; 0.12	3.64	Stabilizing

**Table 3:** Effect of mutation on the 76th amino acid position of nsp8 on the viral replication complex.

Mutation	Chain	Individual ΔΔGStability Prediction (kcal/mol) B; D Chains	ΔΔG Over-All Stability Prediction (kcal/mol)	Effect
S76P	B, D	−1.83; −1.79	−1.39	Destabilizing
S76G	B, D	−1.21; −1.27	−0.66	Destabilizing
S76N	B, D	−0.59; −0.57	−0.23	Destabilizing
S76D	B, D	−1.45; −1.01	−0.14	Destabilizing
S76M	B, D	0.06; 0.63	0	Neutral
S76Q	B, D	−0.3; −0.05	0.11	Stabilizing
S76K	B, D	−0.32; −0.03	0.16	Stabilizing
S76H	B, D	−0.6; −0.02	0.23	Stabilizing
S76T	B, D	−0.5; −0.09	0.32	Stabilizing
S76V	B, D	−0.06; 0.64	0.33	Stabilizing
S76E	B, D	−0.08; 0.19	0.37	Stabilizing
S76L	B, D	−0.02; 0.5	0.43	Stabilizing
S76F	B, D	−0.02; 0.22	0.44	Stabilizing
S76A	B, D	−0.39; −0.01	0.48	Stabilizing
S76W	B, D	−0.13; −0.04	0.55	Stabilizing
S76R	B, D	−0.54; 0.55	0.87	Stabilizing
S76I	B, D	0.09; 1.15	0.99	Stabilizing
S76C	B, D	0.04; 1.0	1.68	Stabilizing
S76Y	B, D	0.64; 1.03	2.27	Stabilizing

**Table 4:** Effect of mutation on the 122nd amino acid position of nsp8 on the viral replication complex.

Mutation	Chain	Individual ΔΔGStability Prediction (kcal/mol) B; D Chains	ΔΔG Over-All Stability Prediction (kcal/mol)	Effect
L122G	B, D	−3.0; −2.09	−2.42	Destabilizing
L122H	B, D	−1.58; −0.74	−1.56	Destabilizing
L122D	B, D	−2.35; −1.35	−1.41	Destabilizing
L122A	B, D	−2.42; −1.58	−1.25	Destabilizing
L122E	B, D	−1.78; −1.08	−1.14	Destabilizing
L122N	B, D	−1.66; −0.97	−0.89	Destabilizing
L122C	B, D	−1.82; −1.32	−0.69	Destabilizing
L122T	B, D	−1.52; −1.36	−0.66	Destabilizing
L122P	B, D	−2.42; −1.34	−0.64	Destabilizing
L122S	B, D	−1.59; −1.08	−0.63	Destabilizing
L122K	B, D	1.11; −0.32	−0.14	Destabilizing
L122V	B, D	−0.02; 0.5	0.06	Stabilizing
L122M	B, D	−0.45; −0.17	0.14	Stabilizing
L122II	B, D	−0.53; −0.27	0.2	Stabilizing
L122Q	B, D	−0.87; −0.41	0.32	Stabilizing
L122F	B, D	−0.33; −0.19	0.66	Stabilizing
L122R	B, D	−0.58; −0.04	0.73	Stabilizing
L122Y	B, D	0.15; 0.18	0.92	Stabilizing
L122W	B, D	−0.35; 0.11	0.95	Destabilizing

**Table 5:** Effect of double mutation on the 121st and 122nd amino acid positions of nsp8 on the viral replication complex.

Mutation	Chain	Individual ΔΔGStability Prediction (kcal/mol) B; D Chains	ΔΔG Over-All Stability Prediction (kcal/mol)	Effect
P121G;L122G	B, D	−2.99; −2.09; −2.75; −0.72	−4.04	Destabilizing
P121D;L122G	B, D	−3.0; −2.16; −2.7; −0.92	−3.98	Destabilizing
P121T;L122G	B, D	−2.99; −2.18; −2.26; −0.7	−3.91	Destabilizing
P121S;L122G	B, D	−2.99; −2.18; −1.66; −0.4	−3.82	Destabilizing
P121N;L122G	B, D	−2.99; −2.16; −2.39; −0.71	−3.69	Destabilizing
P121R	B, D	−1.35; 0.39	1.03	Stabilizing
P121C	B, D	−1.58; 0.1	1.08	Stabilizing
P121E;L122F	B, D	−0.39; 0.89; −1.73; 1.02	1.19	Stabilizing
P121Q	B, D	−1.53; 1.24	1.52	Stabilizing
P121E	B, D	−1.73; 1.18	1.65	Stabilizing

**Table 6:** Comparison of nsp7 mutational effects based on bio-chemoinformatic calculations and wet lab experimental results.

Mutation	Chain	ΔΔGStability Prediction (kcal/mol)	Effect (This Study)	Wet Lab Results Based on Literature [10]
F49A	C	−2.99	Destabilizing	Decreased RdRp efficiency
M52A	C	−2.12	Destabilizing	Decreased RdRp efficiency
L56A	C	−3.09	Destabilizing	Decreased RdRp efficiency
F49A, M52A, L56A	C	−3.46	Destabilizing	Greater decreased RdRp efficiency
C8G	C	−1.97	Destabilizing	Decreased RdRp efficiency
V11A	C	−2.12	Destabilizing	Decreased RdRp efficiency
N37V *	A	0.13	Stabilizing	Not applicable
N37V **	A, C	0.22	Stabilizing	No detrimental effect to nsp7–nsp8 complex
N37V ***	C	−0.15	Destabilizing	Decreased RdRp efficiency

* Nsp7 N37V mutation was introduced into the nsp7–nsp8 dimer complex (PDB: 6YHU). ** Nsp37 N37V mutation was introduced into the nsp7–nsp8 heterotetrameric complex (PDB: 7JLT) of the original wet lab experimental data. *** Nsp7 N37V mutation was introduced into the viral replication protein complex.

**Table 7:** Comparison of nsp8 mutational effects based on bio-chemoinformatic calculations and wet lab experimental results.

Mutation	Chain	Individual ΔΔGStability Prediction (kcal/mol) B; D Chains	ΔΔG Over-All Stability Prediction (kcal/mol)	Effect (This Study)	Wet Lab Results Based on Literature [10]
F92A	B, D	−2.14, −3.09	−3.06	Destabilizing	Decreased RdRp efficiency
M90A	B, D	−1.92, −2.48	−1.39	Destabilizing	Decreased RdRp efficiency
M94A	B, D	−1.18, −2.84	−1.94	Destabilizing	Decreased RdRp efficiency

## Data Availability

Data are contained within the article and Supplementary Materials.

## Supplementary Materials

The following supporting information can be downloaded at: https://www.mdpi.com/article/10.3390/cimb46030165/s1, Supplementary Figure S1. Experimental workflow of the study. Supplementary Figure S2. Multiple sequence alignment for nsp7 (A) and nsp8 (B) during conserved domain database (CDD) analysis. Supplementary Dataset S1. Reference sequences of nsp7 and nsp8 for Conserve Domain Database (CDD) Analysis. Supplementary Dataset S2. Native protein sequences of nsp12, nsp7 and nsp8. Supplementary Dataset S3. Temporal mutation of nsp7 and nsp8 (2022- 2023). Supplementary Dataset S4. Hydrophobic interactions in threonine to isoleucine mutation of nsp8. Supplementary Dataset S5. Double mutation analysis for P121X, L122X. Supplementary Dataset S6. Non-covalent interactions for critical interaction residues of nsp7. Supplementary Dataset S7. Non-covalent interactions for critical interaction residues of nsp8. Supplementary Dataset S8. Computational alanine scan of the viral replication complex. Supplementary Dataset S9. Statistics of amino acid contributions to viral replication protein complex stability. Supplementary Dataset S10. Hydrogen bonding interactions in the potential hotspot residues in nsp8.
