# Supplementary material for: Bio-Chemoinformatics-Driven Analysis of nsp7 and nsp8 Mutations and Their Effects on Viral Replication Protein Complex Stability
Source: Curr Issues Mol Biol. 2024 Mar 18;46(3):2598–619. doi: 10.3390/cimb46030165 (PMC10968879; doi:10.3390/cimb46030165)
Supplement: Supplementary file 1 [file cimb-46-00165-s001.zip › Supplementary Dataset S1. Reference sequences of nsp7 and nsp8 for Conserve Domain Database (CDD) Analysis.pdf]

Supplementary Dataset S1. Reference sequences of nsp7 and nsp8 for Conserve Domain Database (CDD) Analysis.

NCBI Reference (Nsp7)	Description
6NUR_C	Severe acute respiratory syndrome-related coronavirus
1YSY_A	Severe acute respiratory syndrome-related coronavirus
2AHM_A	SARS coronavirus
6WIQ_A	Severe acute respiratory syndrome coronavirus 2
6WTC_C	Severe acute respiratory syndrome coronavirus 2
7BV2_C	Severe acute respiratory syndrome coronavirus 2
AAP33696	SARS coronavirus Frankfurt 1
ADY69163	Zaria bat coronavirus
YP_009072438	Bat Hp-betacoronavirus/Zhejiang2013
P0C6T6	Rousettus bat coronavirus HKU9
AOG30811	Rousettus bat coronavirus
AWV67038	Bat coronavirus
AGT28265	Betacoronavirus Erinaceus/VMC/DEU/2012
QGA70691	Erinaceus hedgehog coronavirus HKU31
AUM60023	Middle East respiratory syndrome-related coronavirus
P0C6W4	Pipistrellus bat coronavirus HKU5
BBJ36006	Bat coronavirus

ASL68951	Hypsugo bat coronavirus HKU25
AVV62535	Middle East respiratory syndrome-related coronavirus
P0C6W3	Tylonycteris bat coronavirus HKU4
K9N7C7	Betacoronavirus England 1
ANA96038	Bat coronavirus
P0C6W9	Bovine coronavirus Mebus
P0C6X4	Human coronavirus HKU1 (isolate N5)
ATP66731	Rodent coronavirus
P0C6V0	Murine hepatitis virus strain A59

NCBI Reference (Nsp8)	Description
2AHM_E	SARS coronavirus
7BV1_B	Severe acute respiratory syndrome-related coronavirus
6NUR_B	Severe acute respiratory syndrome-related coronavirus
6NUS_B	Severe acute respiratory syndrome-related coronavirus
7BV2_B	Severe acute respiratory syndrome-related coronavirus
2AHM_H	SARS coronavirus
NP_828850	Severe acute respiratory syndrome-related coronavirus
P0C6V9	Bat CoV 279/2005

P0C6W2	Bat SARS coronavirus HKU3
P0C6F7	Bat coronavirus (BtCoV/133/2005)
P0C6T4	Tylonycteris bat coronavirus HKU4
P0C6T5	Pipistrellus bat coronavirus HKU5
P0C6T6	Rousettus bat coronavirus HKU9
P0C6U3	Human coronavirus HKU1 (isolate N1)
P0C6V0	Murine hepatitis virus strain A59
AIG13097	Coronavirus Neoromicia/PML-PHE1/RSA/2011
YP_009072438	Bat Hp-betacoronavirus/Zhejiang2013
AJA91214	Betacoronavirus HKU24
AOG30811	Rousettus bat coronavirus
ASL68952	Hypsugo bat coronavirus HKU25
ATP66760	Rodent coronavirus
AUM60013	Middle East respiratory syndrome-related coronavirus
AVV62524	Middle East respiratory syndrome-related coronavirus
AWV67046	Bat coronavirus
QCC20711	Hedgehog coronavirus 1
QEI22730	Betacoronavirus BtCoV22/29
BBJ36007	Bat coronavirus
QGA70691	Erinaceus hedgehog coronavirus HKU31
AAP33696	SARS coronavirus Frankfurt 1
