# Supplementary material for: Bio-Chemoinformatics-Driven Analysis of nsp7 and nsp8 Mutations and Their Effects on Viral Replication Protein Complex Stability
Source: Curr Issues Mol Biol. 2024 Mar 18;46(3):2598–619. doi: 10.3390/cimb46030165 (PMC10968879; doi:10.3390/cimb46030165)
Supplement: Supplementary file 1 [file cimb-46-00165-s001.zip › Supplementary Dataset S10. Hydrogen bonding interactions in the potential hotspot residues in nsp8.pdf]

Supplementary Dataset S10. Hydrogen bonding interactions in the potential hotspot residues in nsp8.

Trp-154 (B W1086A; D W1367A):

B-chain:

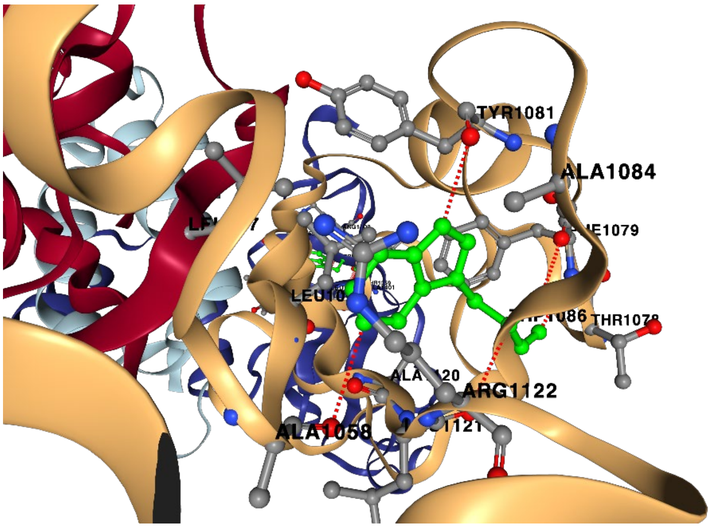

D-chain:

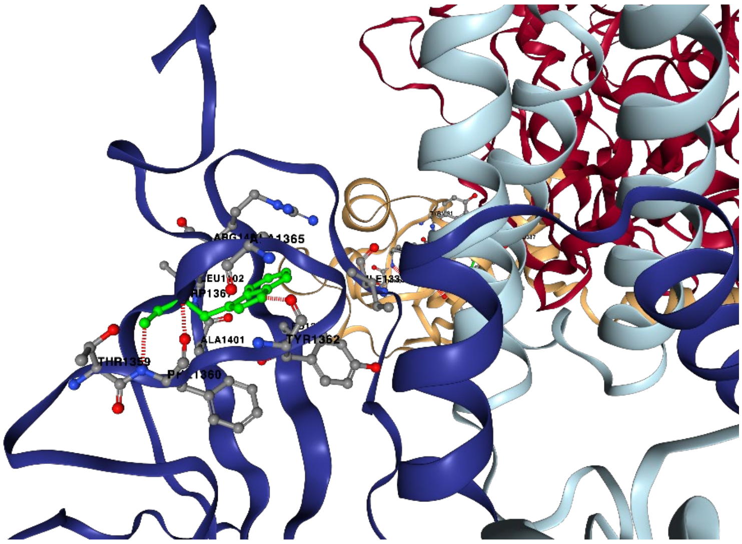

Tyr-135 (B Y1067A; D Y1348A)

B-chain and D-chain:

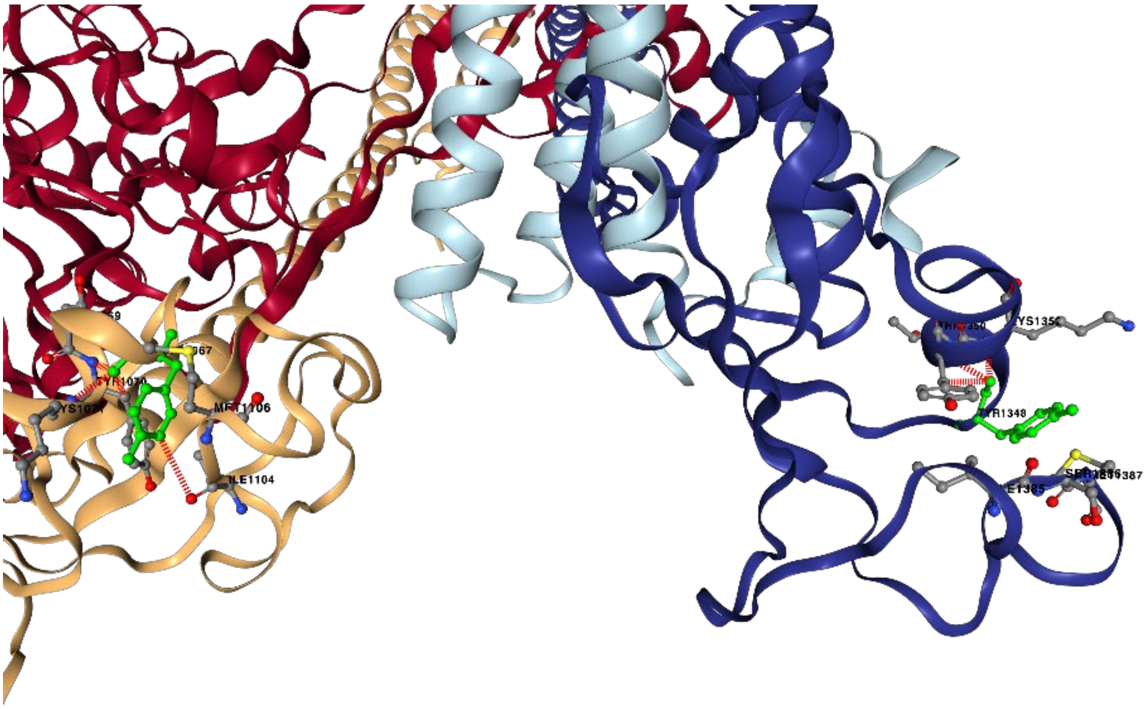

Phe-15 (B F947A; D 1228A)

B-chain and D-chain:

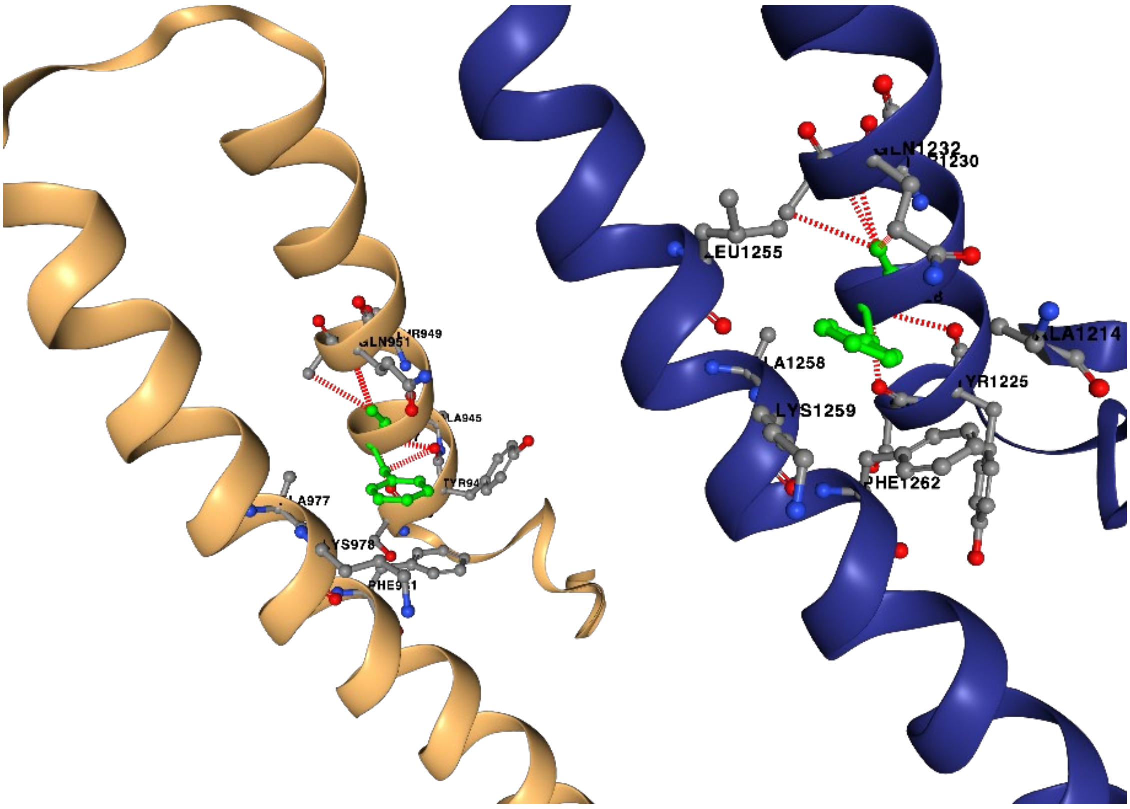

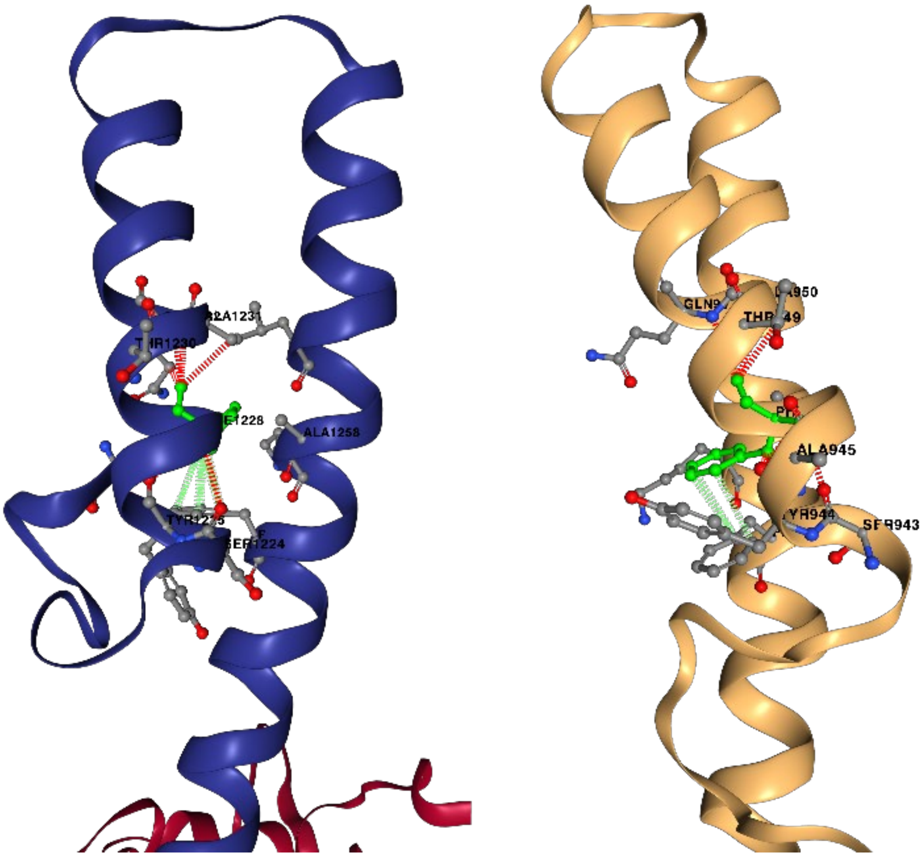

Note: Light-green dotted lines represent aromatic interaction.
