# Supplementary material for: Bio-Chemoinformatics-Driven Analysis of nsp7 and nsp8 Mutations and Their Effects on Viral Replication Protein Complex Stability
Source: Curr Issues Mol Biol. 2024 Mar 18;46(3):2598–619. doi: 10.3390/cimb46030165 (PMC10968879; doi:10.3390/cimb46030165)
Supplement: Supplementary file 1 [file cimb-46-00165-s001.zip › Supplementary Dataset S2. Native protein sequences of nsp12, nsp7 and nsp8.pdf]

Supplementary Dataset S2. Native protein sequences of nsp12, nsp7 and nsp8.

nsp7 [Severe acute respiratory syndrome coronavirus 2]

NCBI Reference Sequence: YP_009725303.1

>YP_009725303.1 nsp7 [Severe acute respiratory syndrome coronavirus 2]
SKMSDVKCTSVVLLSVLQQLRVESSSKLWAQCVQLHNDILLAKDTTEAFEKMVSLLSV
LLSMQGAVDINKLCEEMLDNRATLQ

nsp8 [Severe acute respiratory syndrome coronavirus 2]

NCBI Reference Sequence: YP_009725304.1

>YP_009725304.1 nsp8 [Severe acute respiratory syndrome coronavirus 2]
AIASEFSSLPSYAAFATAQEAYEQAVANGDSEVVLKKLKKSLNVAKSEFDRDAAMQRK
LEKMADQAMTQMYKQARSEDKRAKVTSAMQTMLFTMLRKLDNDALNNIINNARDGCV
PLNIIPLTTAAKLMVVIPDYNTYKNTCDGTTFTYASALWEIQQVVDADSKIVQLSEISMDN
SPNLAWPLIVTALRANSAVKLQ

nsp12 RNA-dependent RNA polymerase [Severe acute respiratory syndrome coronavirus 2]

NCBI Reference Sequence: YP_009725307.1

>YP_009725307.1 RNA-dependent RNA polymerase [Severe acute respiratory syndrome coronavirus 2]
SADAQSFLNRVCGVSAARLTPCGTGTSTDVVYRAFDIYNDKVAGFAKFLKTNCCRFQE
KDEDDNLIDSYFVVKRHTFSNYQHEETIYNLLKDCPAVAKHDFFKFRIDGDMVPHISRQ
RLTKYTMADLVYALRHFDEGNCDTLKEILVTYNCCDDDYFNKKDWYDFVENPDILRVY
ANLGERVRQALLKTVQFCDAMRNAGIVGVLTLDNQDLNGNWYDFGDFIQTTPGSGVP
VVDSYYSLLMPILTLTRALTAESHVDTDLTKPYIKWDLLKYDFTEERLKLFDRYFKYWD
QTYHPNCVNCLDDRCILHCANFNVLFSTVFPPTSFGPLVRKIFVDGVPFVVSTGYHFRE
LGVVHNQDVNLHSSRLSFKELLVYAADPAMHAASGNLLLDKRTTCFSVAALTNNVAFQ
TVKPGNFNKDFYDFAVSKGFFKEGSSVELKHFFFAQDGNAAISDYDYYRYNLPTMCDI
RQLLFVVEVVDKYFDCYDGGCINANQVIVNNLDKSAGFPFNKWGKARLYYDSMSYED
QDALFAYTKRNVIPTITQMNLKYAISAKNRARTVAGVSICSTMTNRQFHQKLLKSIAATR
GATVVIGTSKFYGGWHNMLKTVYSDVENPHLMGWDYPKCDRAMPNMLRIMASLVLA
RKHTTCCSLSHRFYRLANECAQVLSEMVMCGGSLYVKPGGTSSGDATTAYANSVFNI
CQAVTANVNALLSTDGNKIADKYVRNLQHRLYECLYRNRDVDTDFVNEFYAYLRKHFS
MMILSDDAVVCFNSTYASQGLVASIKNFKSVLYYQNNVFMSEAKCWTETDLTKGPHEF
CSQHTMLVKQGDDYVYLPYPDPSRILGAGCFVDDIVKTDGTLMIERFVSLAIDAYPLTK
HPNQEYADVFHLYLQYIRKLHDELTGHMLDMYSVMLTNDNTSRYWEPEFYEAMYTPH
TVLQ
