# Supplementary material for: Bio-Chemoinformatics-Driven Analysis of nsp7 and nsp8 Mutations and Their Effects on Viral Replication Protein Complex Stability
Source: Curr Issues Mol Biol. 2024 Mar 18;46(3):2598–619. doi: 10.3390/cimb46030165 (PMC10968879; doi:10.3390/cimb46030165)

Supplementary Dataset S4. Hydrophobic interactions in threonine to isoleucine mutation of nsp8

T145I:

Native structure (B-chain)

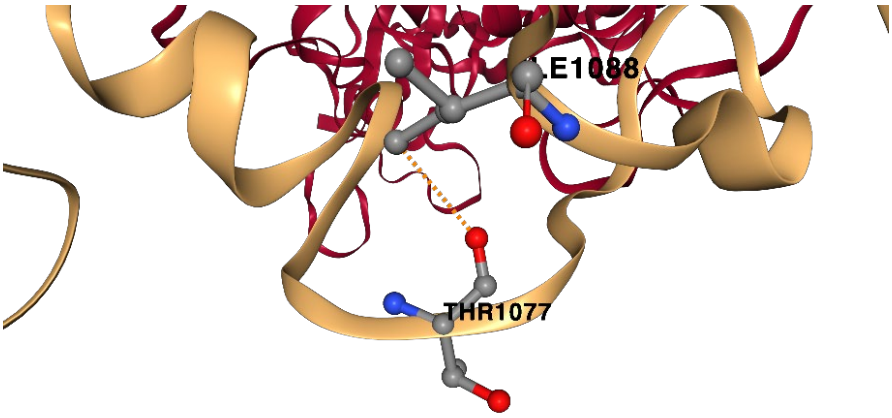

Mutant structure (B-chain)

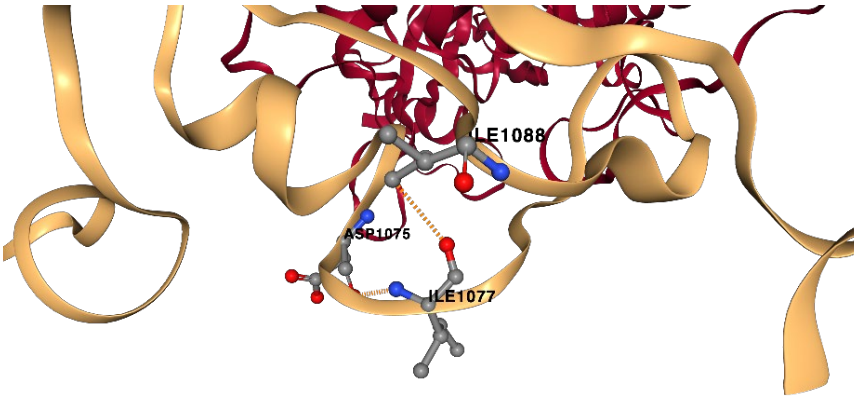

T148I

Native structure (B-chain)

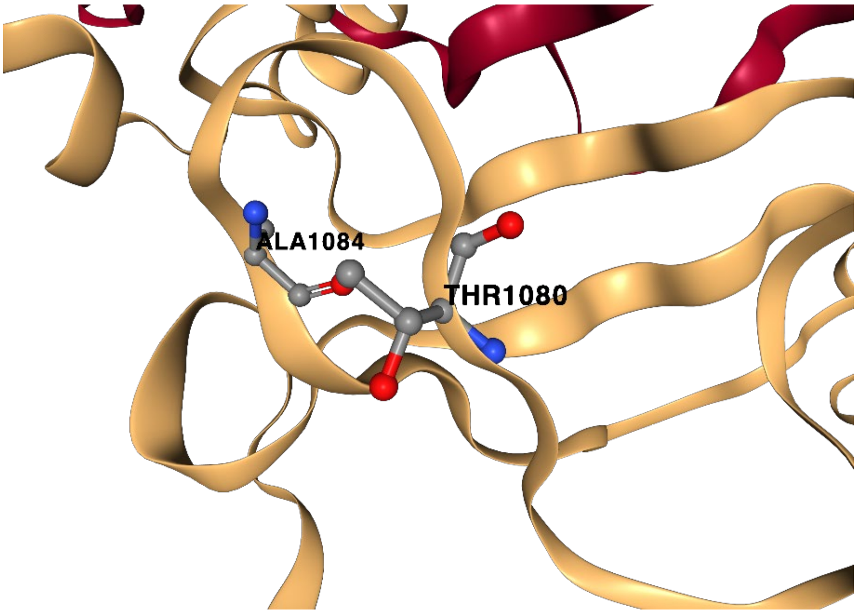

Mutant structure (D-chain)

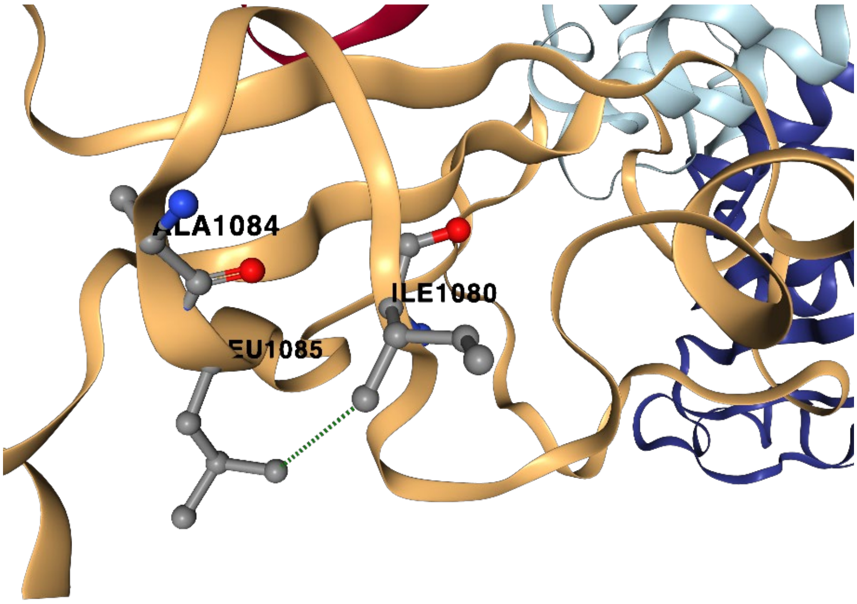

Native structure (D-chain)

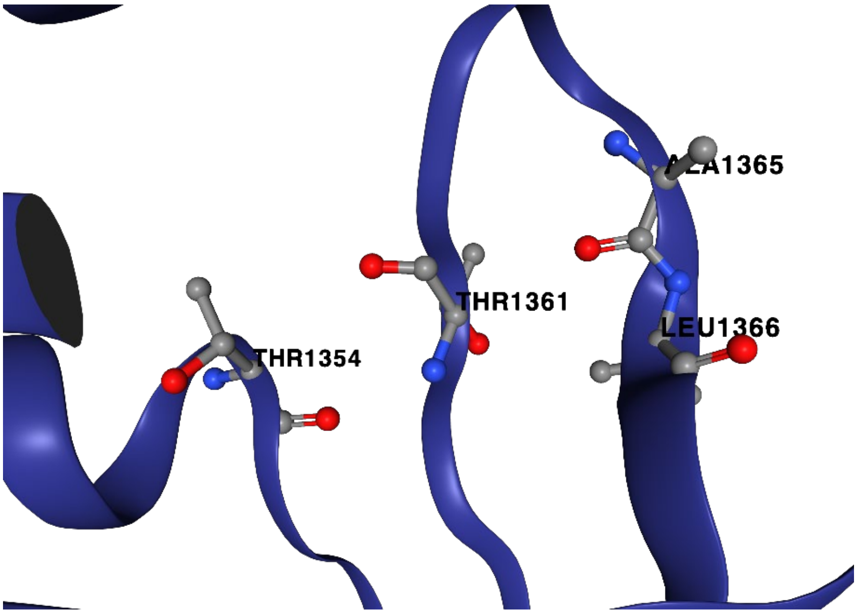

Mutant structure (D-chain)

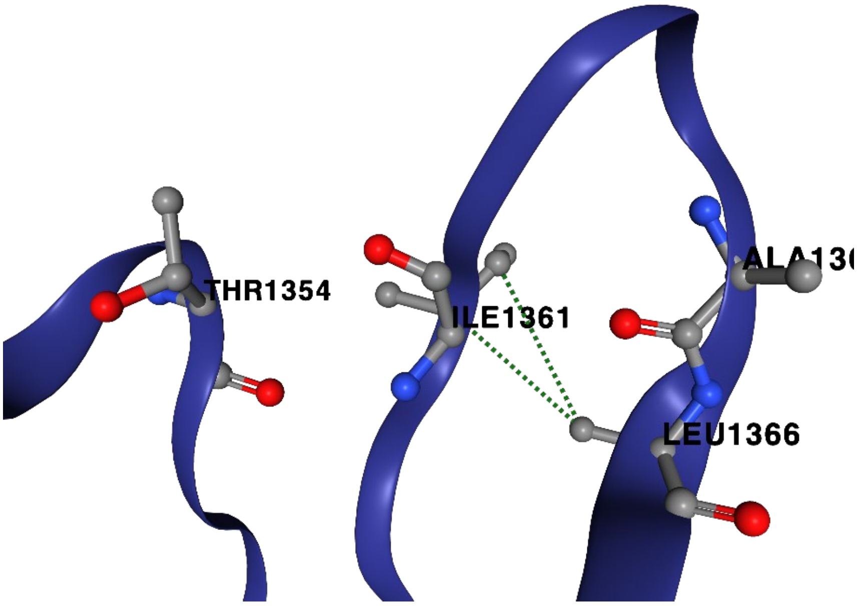

T123I

Native structure (B-chain)

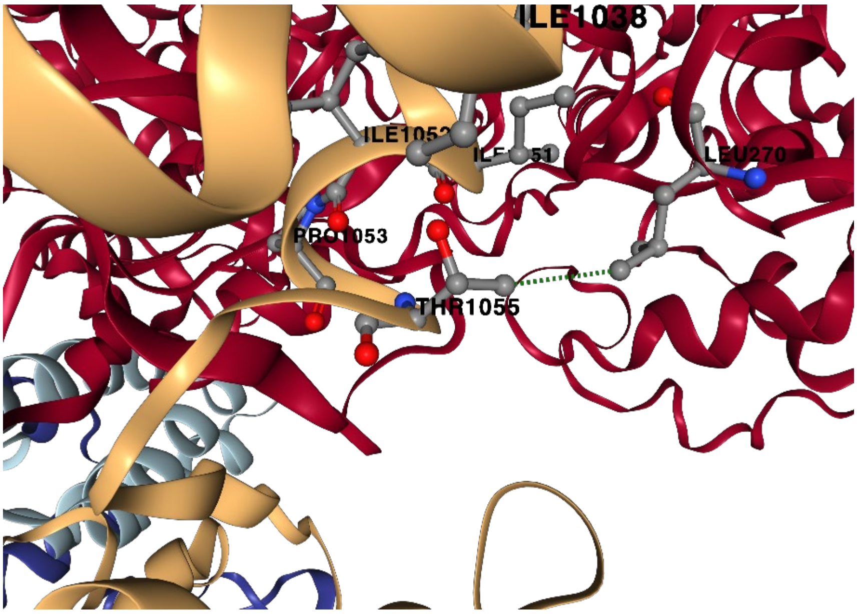

Mutant structure (B-chain)

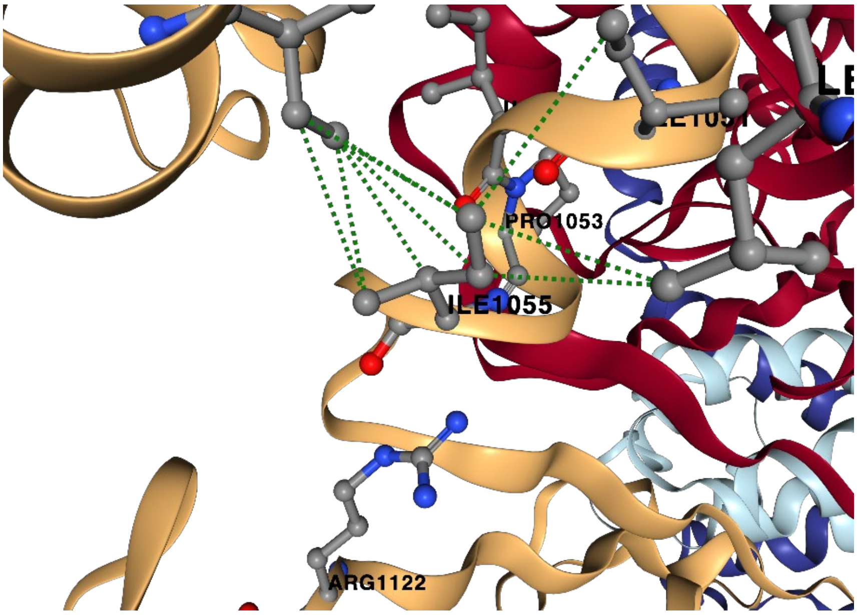

T187I

Native structure (B-chain)

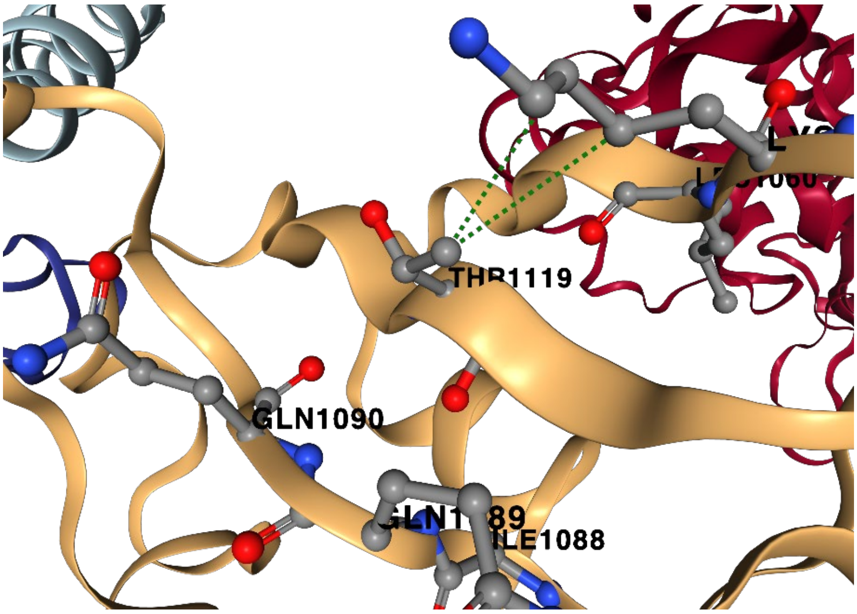

Mutant structure (B-chain)

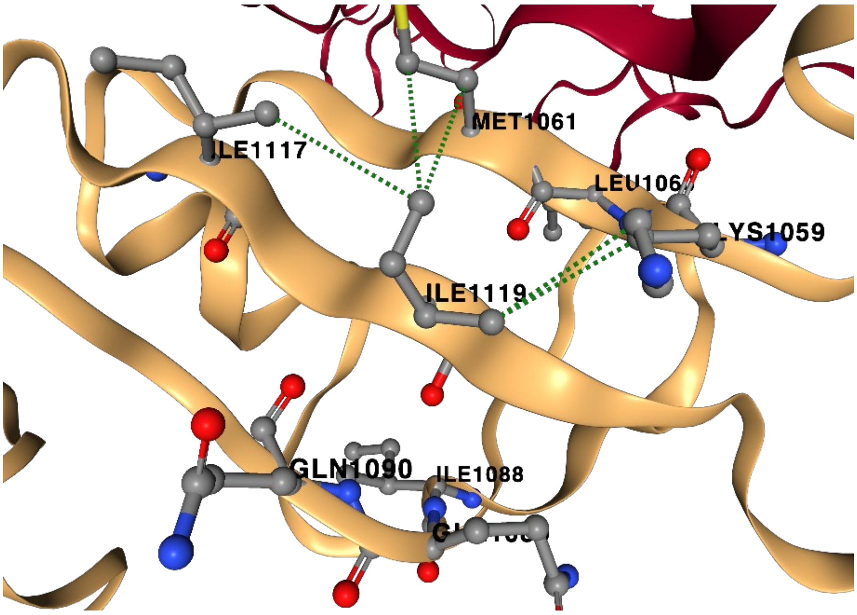

Supplement: Supplementary file 1 [file cimb-46-00165-s001.zip › Supplementary Dataset S4. Hydrophobic interactions in threonine to isoleucine mutation of nsp8.pdf]
