# Supplementary material for: Bio-Chemoinformatics-Driven Analysis of nsp7 and nsp8 Mutations and Their Effects on Viral Replication Protein Complex Stability
Source: Curr Issues Mol Biol. 2024 Mar 18;46(3):2598–619. doi: 10.3390/cimb46030165 (PMC10968879; doi:10.3390/cimb46030165)
Supplement: Supplementary file 1 [file cimb-46-00165-s001.zip › Supplementary Dataset S6. Non-covalent interactions for critical interaction residues of nsp7.pdf]

Supplementary Dataset S6. Non-covalent interactions for critical interaction residues of nsp7.

K7 (WT)

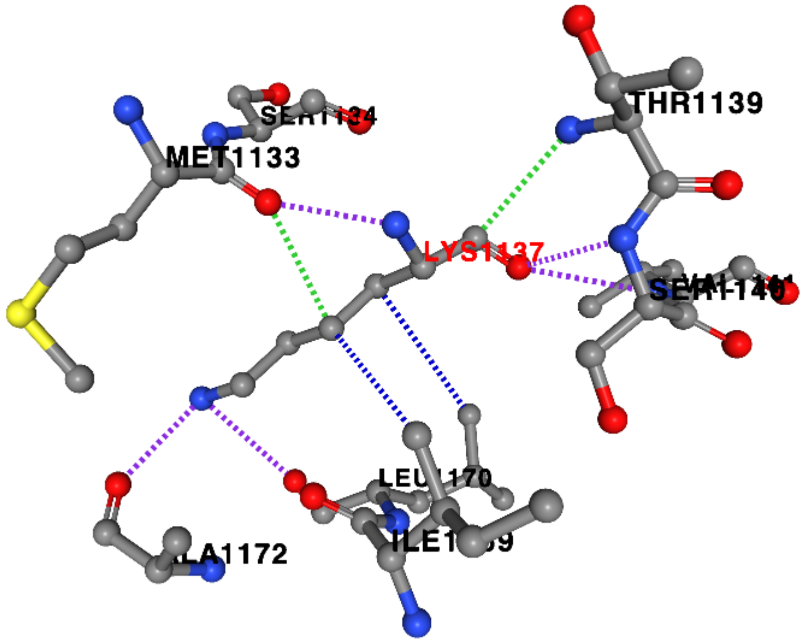

K7L

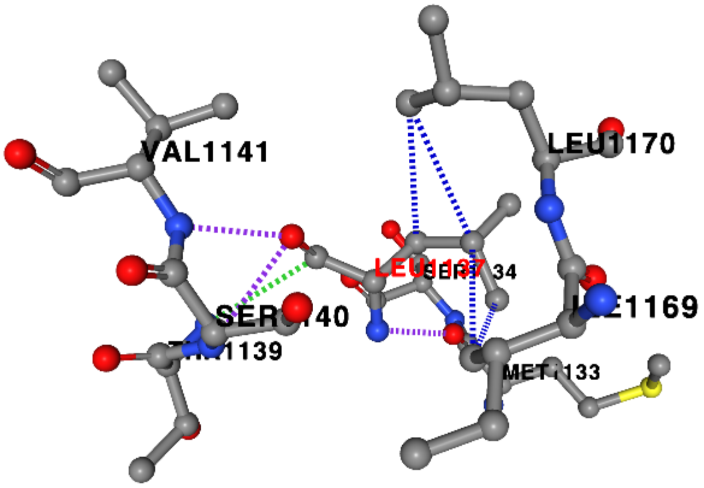

K7F

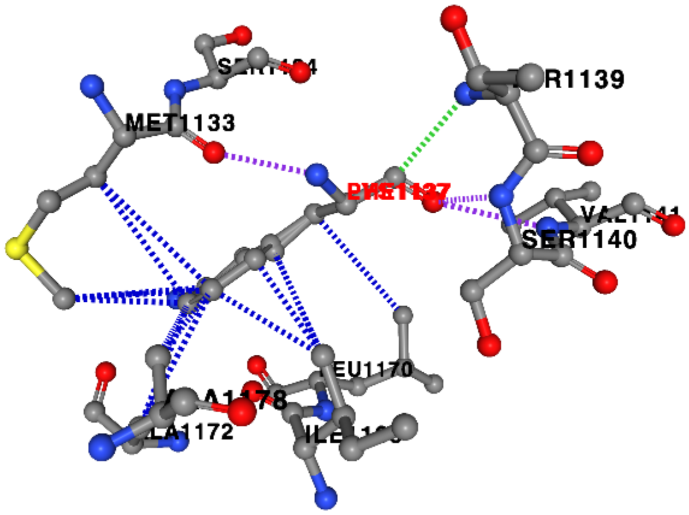
MET1133
SER1140

K7I

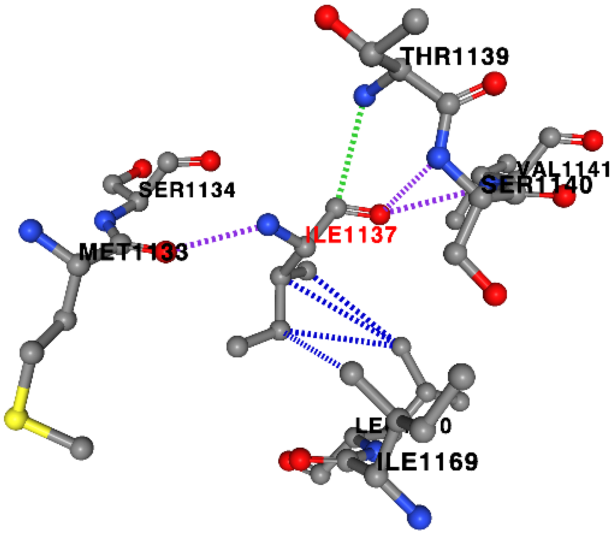
THR1139
SER1134
MET1133
ILE1137
VAL1141
SER1140
ILE1169

K7D

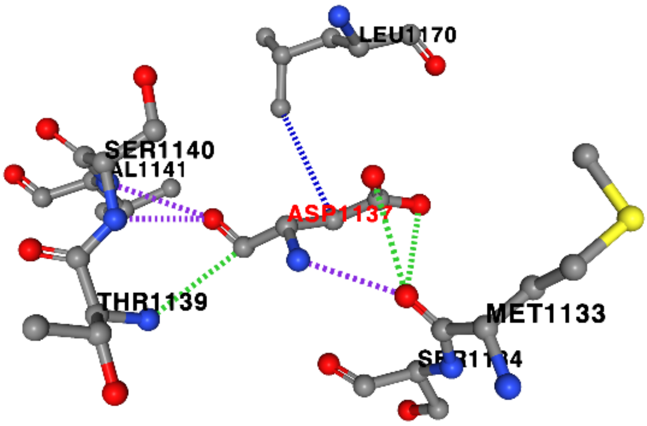
LEU1170
SER1140
ASP1137
THR1139
MET1133

K7P

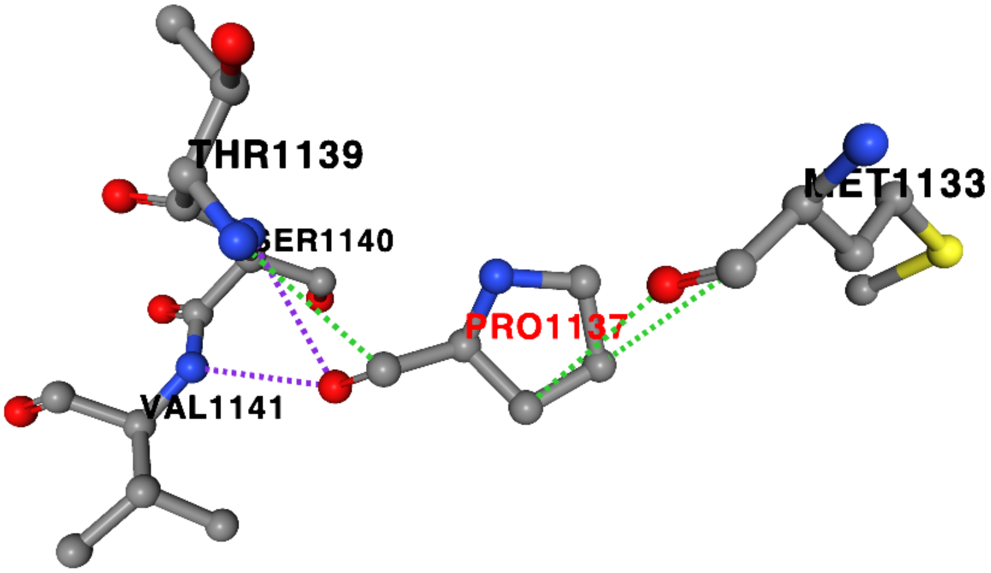
THR1139
PRO1137
VAL1141

K7G

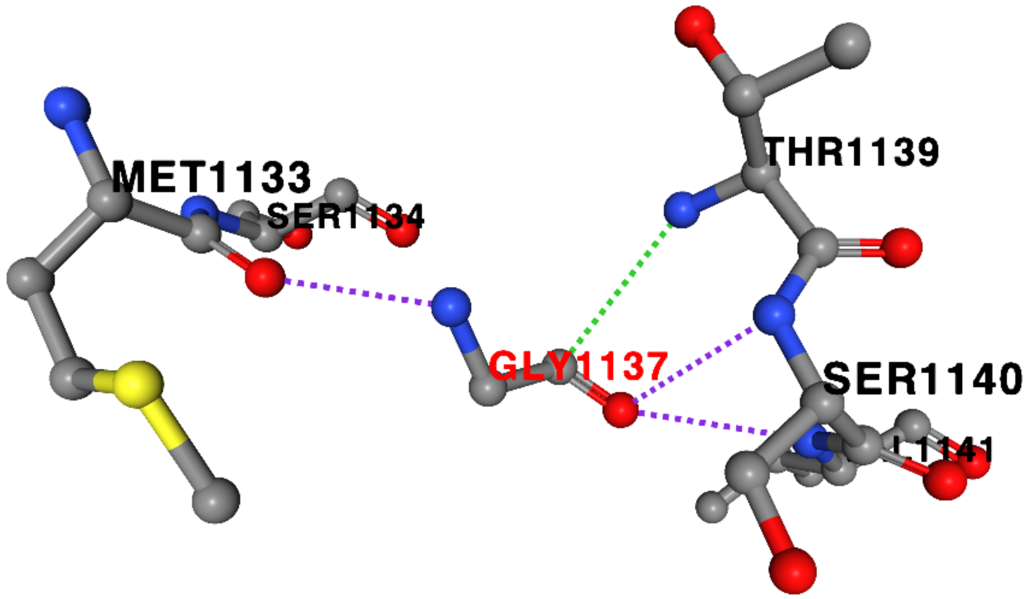
MET1133
SER1134
THR1139
GLY1137
SER1140

H36 (WT)

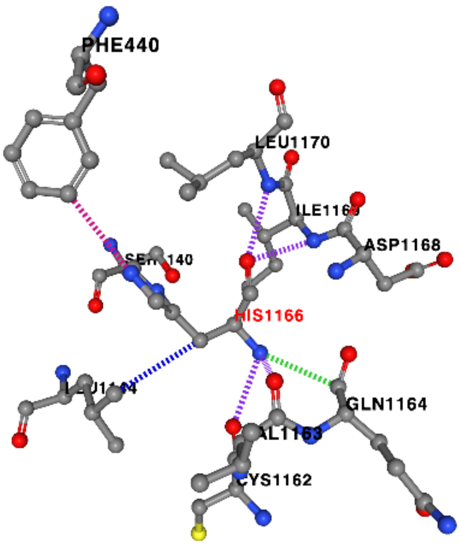
PHE440
LEU1170
ASP1168
HIS1166
GLN1164
CYS1162

H36Y

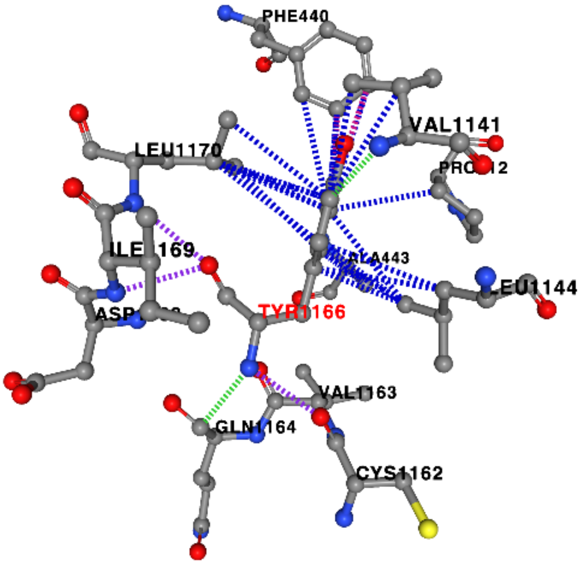
PHE440
VAL1141
LEU1170
ILE1169
LEU1144
TYR1166
VAL1163
GLN1164
CYS1162

H36L

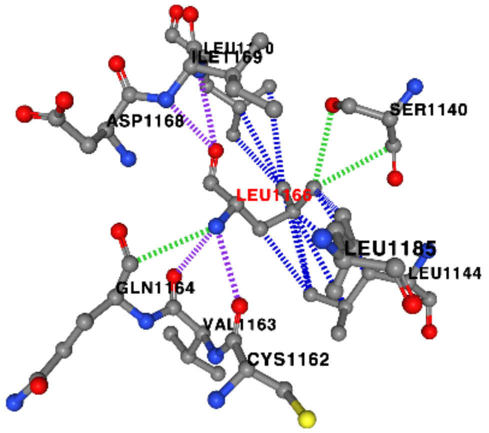
ILE1169
SER1140
ASP1168
LEU1185
LEU1144
GLN1164
VAL1163
CYS1162

H36F

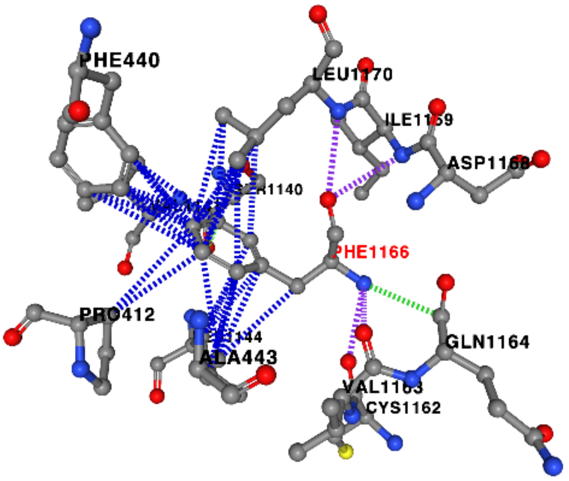
PHE440
LEU1170
ASP1168
PHE1166
PRO412
GLN1164
ALA443
CYS1162

H36R

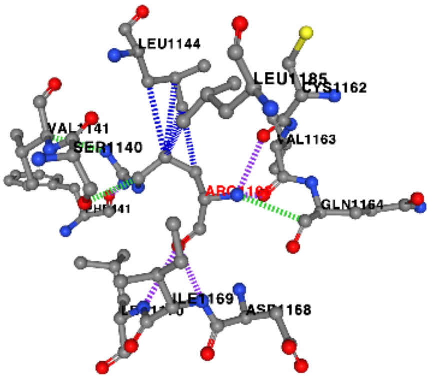
LEU1144
LEU1185
CYS1162
SER1140
VAL1163
ILE1169
ASP1168

H36G

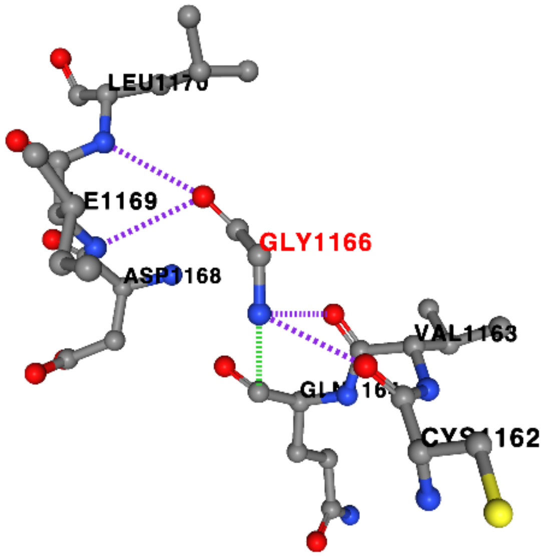
LEU1170
E1169
GLY1166
ASP1168
VAL1163
CYS1162

H36Q

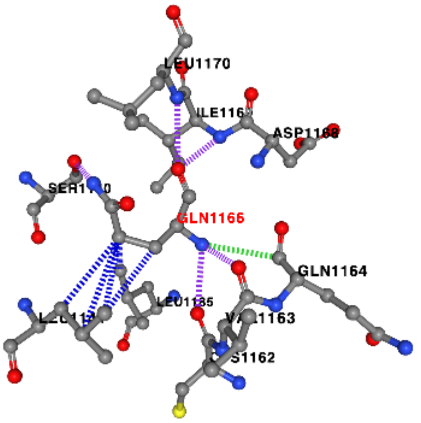
LEU1170
ASP1168
GLN1166
GLN1164
VAL1163

N37 (WT)

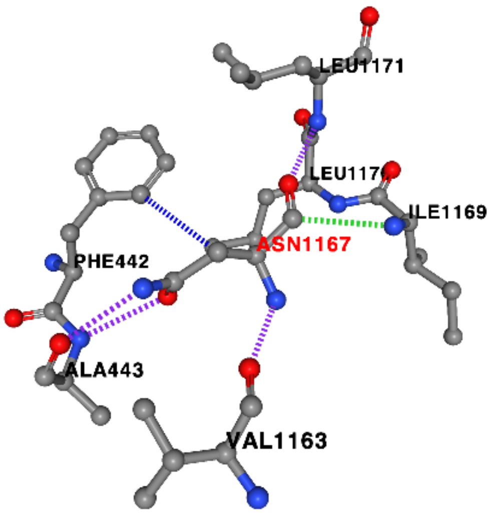
LEU1171
ILE1169
ASN1167
PHE442
ALA443
VAL1163

N37L

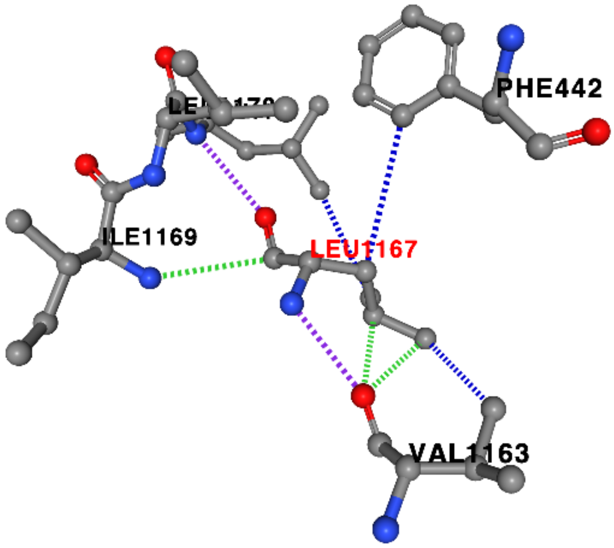
PHE442
ILE1169
LEU1167
VAL1163

N37I

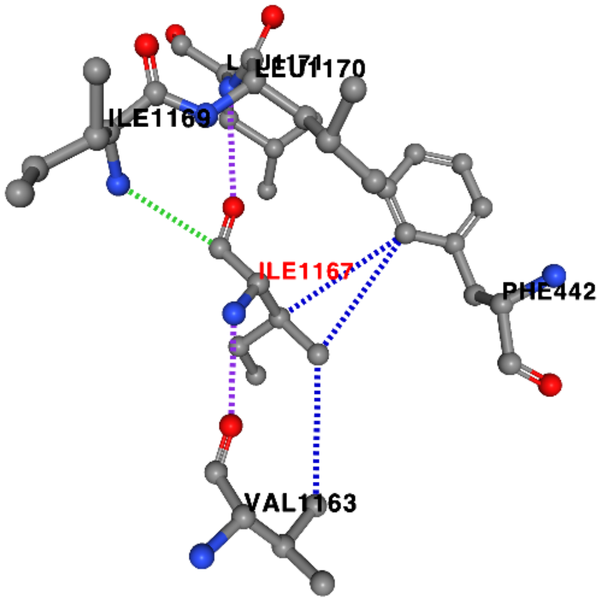
LEU1170
ILE1169
ILE1167
PHE442
VAL1163

N37L

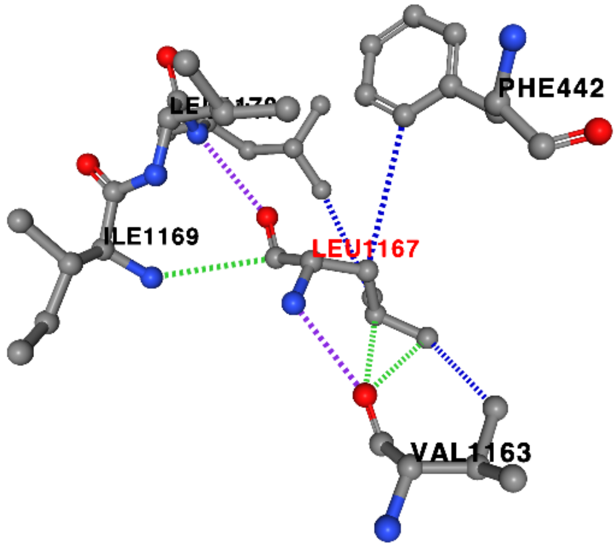
PHE442
ILE1169
LEU1167
VAL1163

N37P

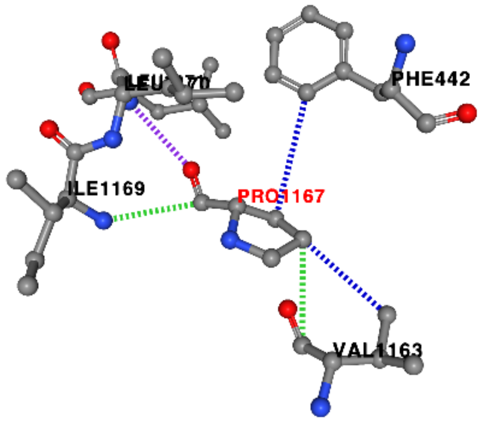

N37G

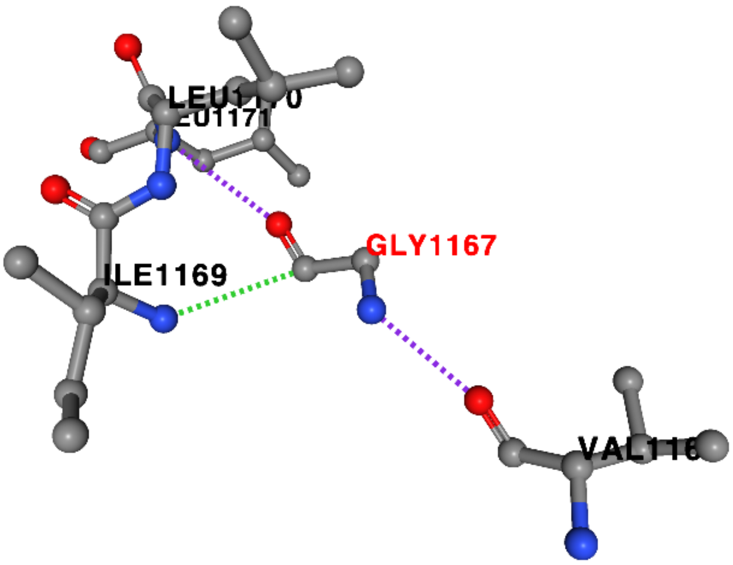

N37K

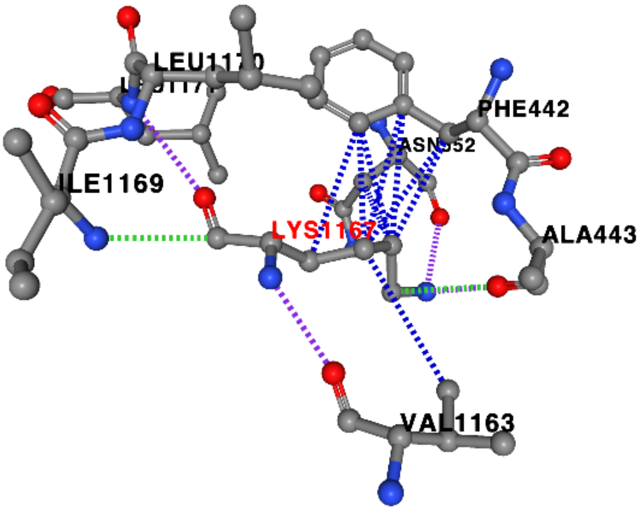

Legend:

Hydrogen Bond	Aromatic	Polar	Ionic
Hydrophobic	Carbonyl	Clash	VDW
