# Supplementary material for: Bio-Chemoinformatics-Driven Analysis of nsp7 and nsp8 Mutations and Their Effects on Viral Replication Protein Complex Stability
Source: Curr Issues Mol Biol. 2024 Mar 18;46(3):2598–619. doi: 10.3390/cimb46030165 (PMC10968879; doi:10.3390/cimb46030165)
Supplement: Supplementary file 1 [file cimb-46-00165-s001.zip › Supplementary Dataset S7. Non-covalent interactions for critical interaction residues of nsp8.pdf]

Supplementary Dataset S7. Non-covalent interactions for critical interaction residues of nsp8.

K58 B-chain (WT):

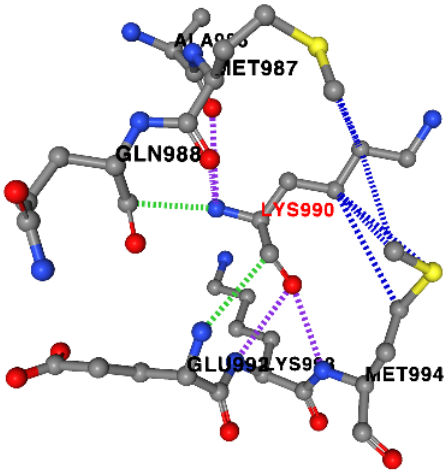

K58 D-chain (WT):

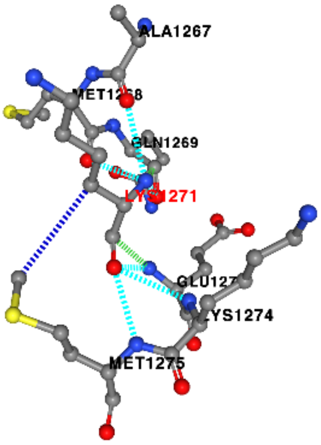

K58I B-chain

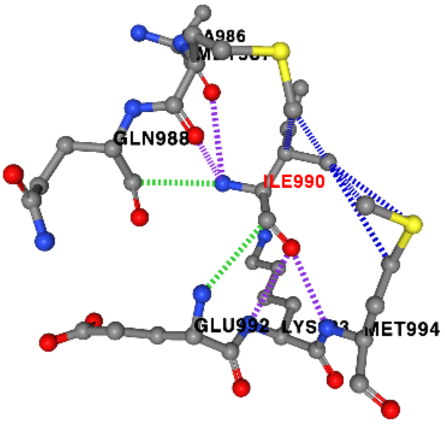

K58I D-chain

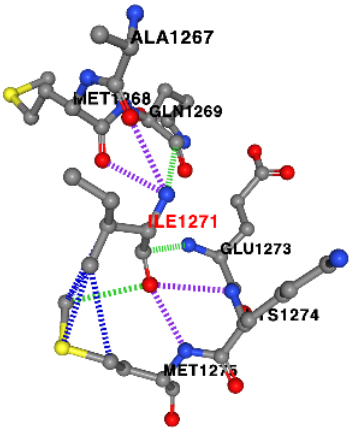

K58L B-chain:

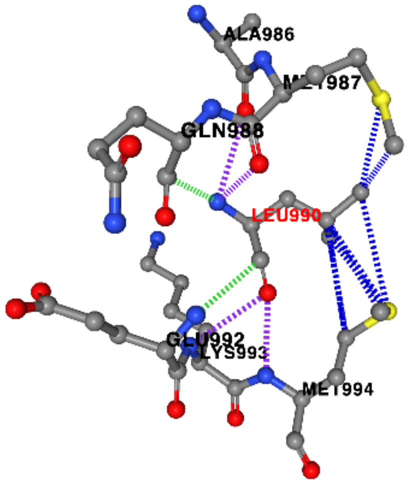

K58L D-chain:

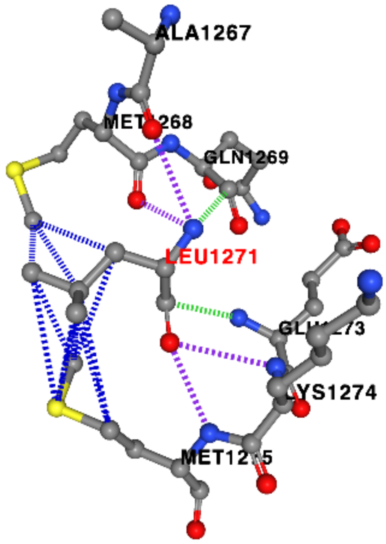

K58Q B-chain:

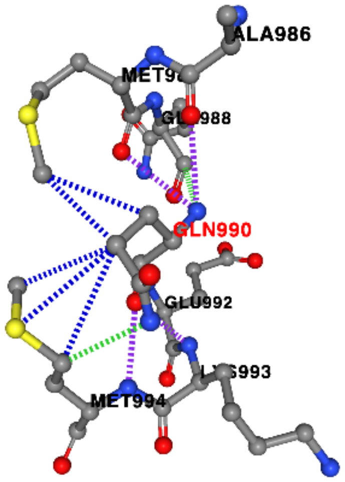

K58Q D-chain:

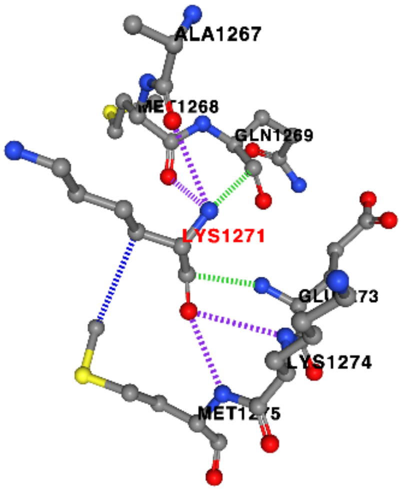

K58G B-chain:

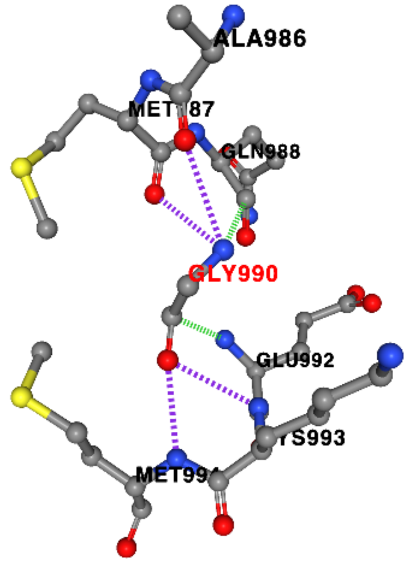

K58G D-chain:

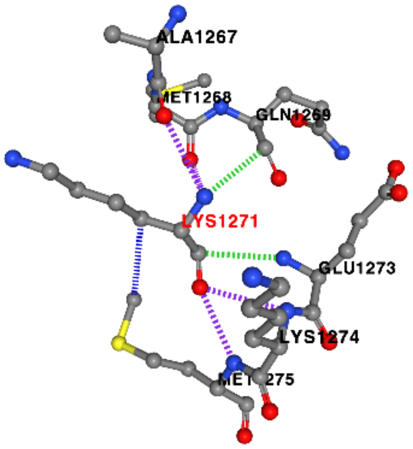

P183 B-chain (WT):

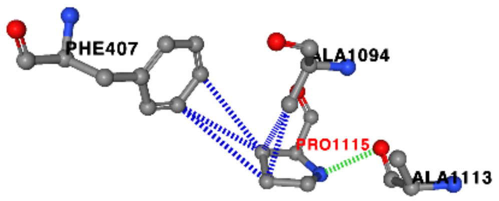

P183 D-chain (WT):

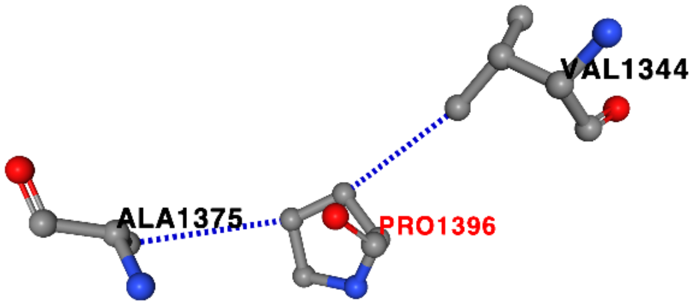

P183C B-chain:

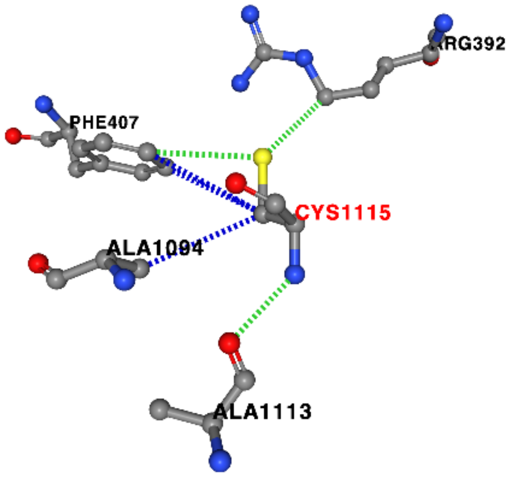

P183C D-chain:

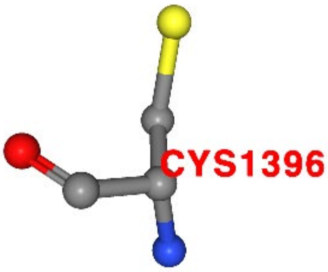

P183V B-chain:

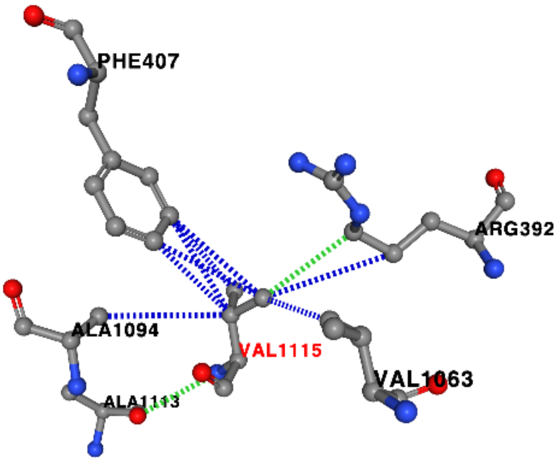

P183V D-chain:

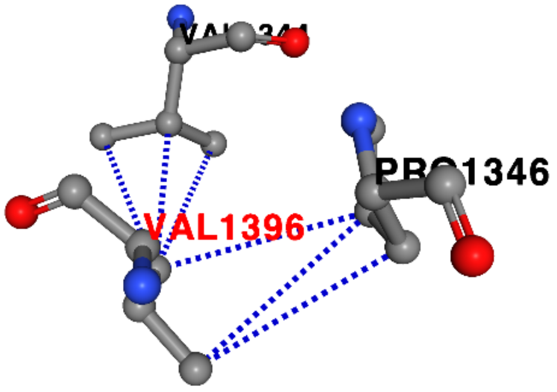

P183Y B-chain:

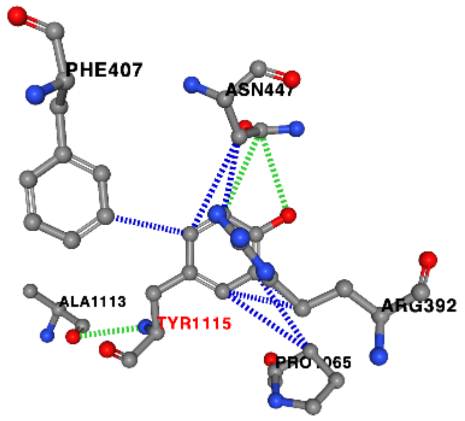

P183Y D-chain:

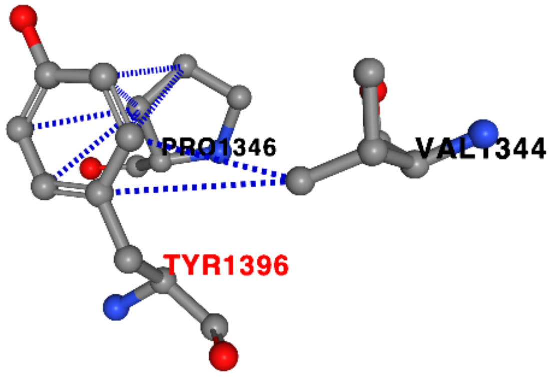

P183F B-chain:

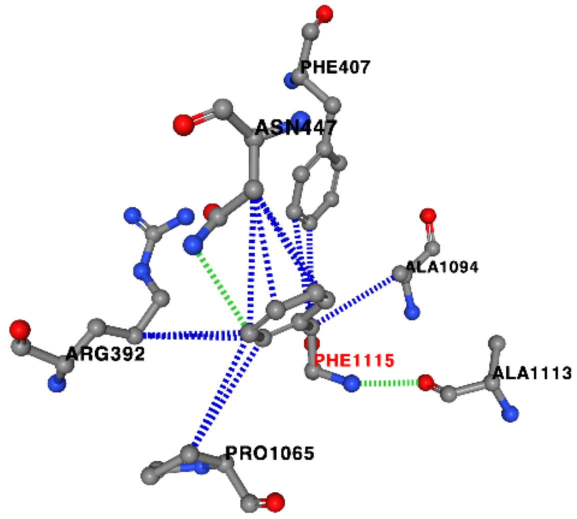

P183F D-chain:

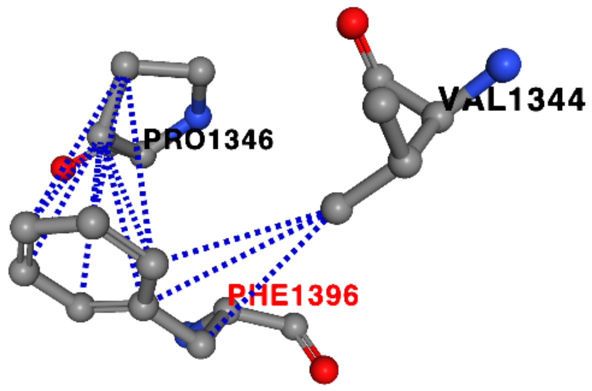

R190

R190 B-chain (WT):

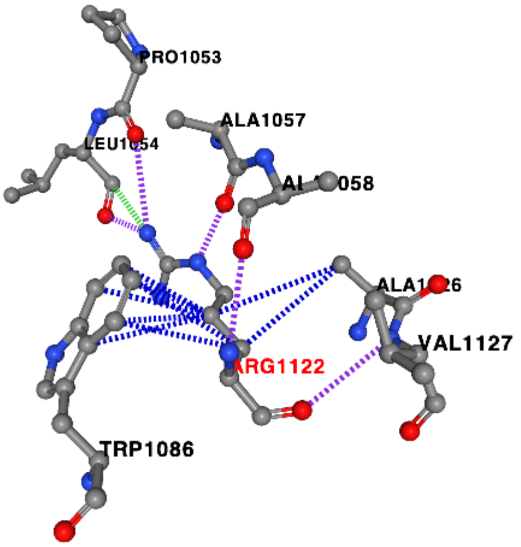

R190 D-chain (WT):

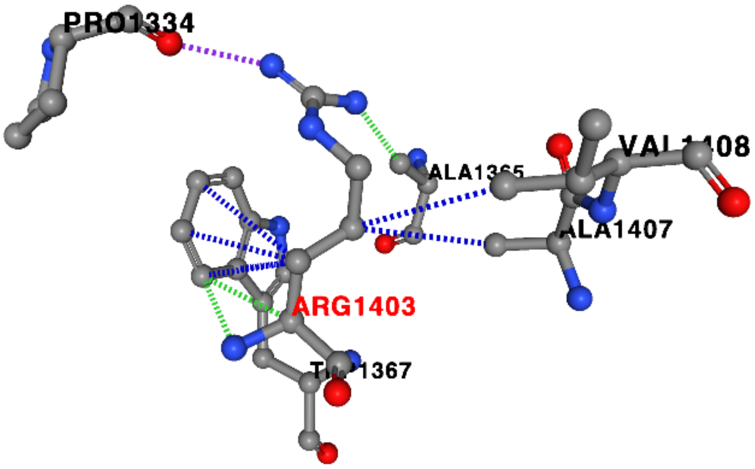

R190L B-chain:

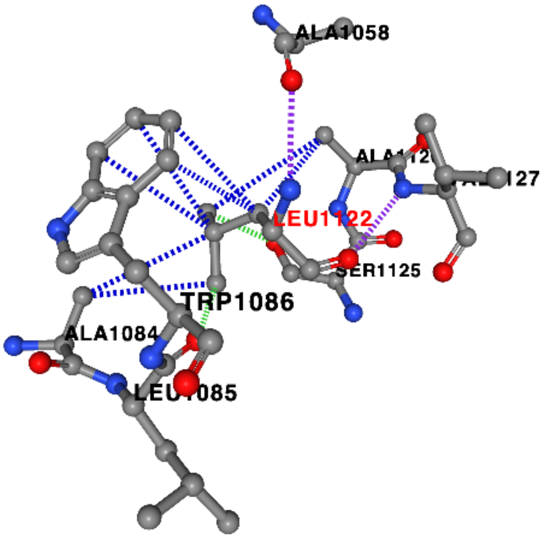

R190L D-chain:

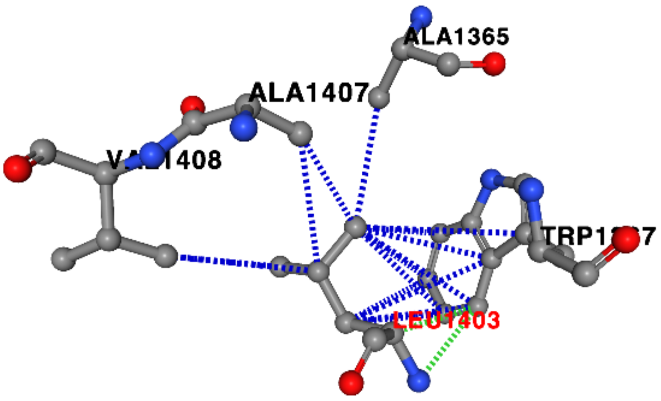

R190M B-chain:

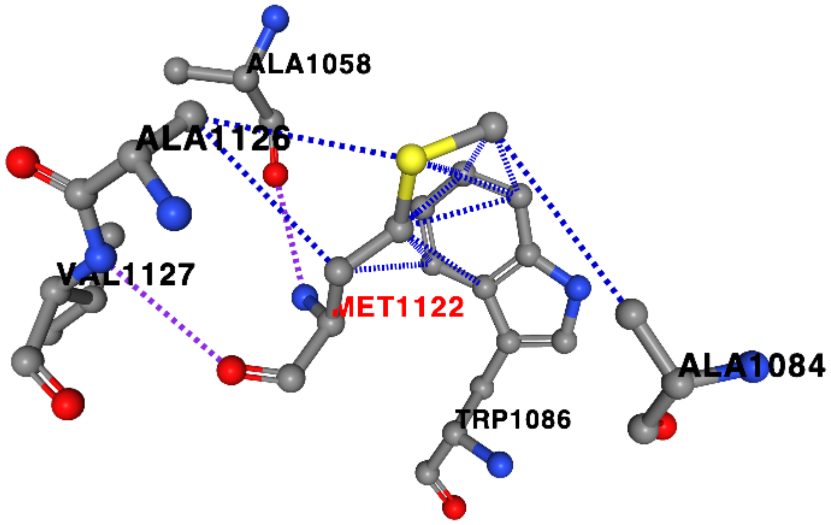

R190M D-chain:

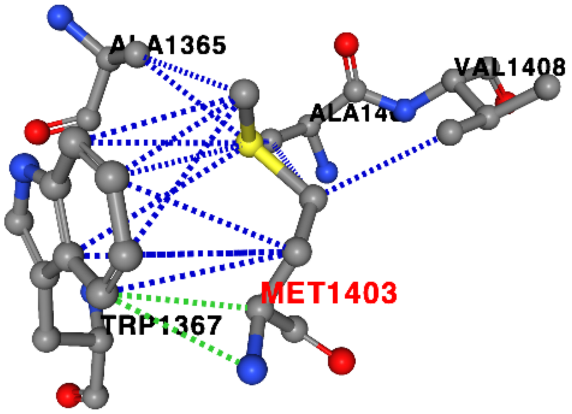

R190S B-chain:

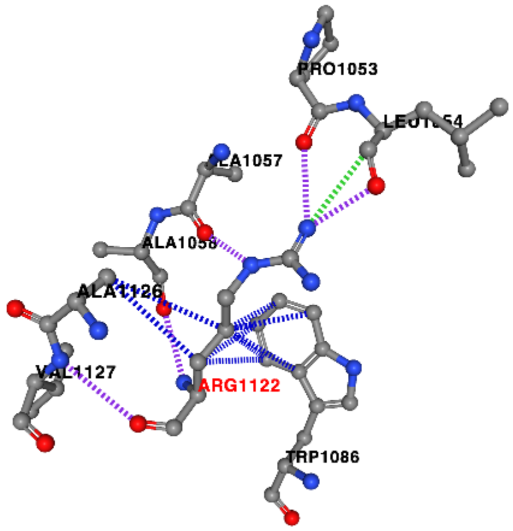

R190S D-chain:

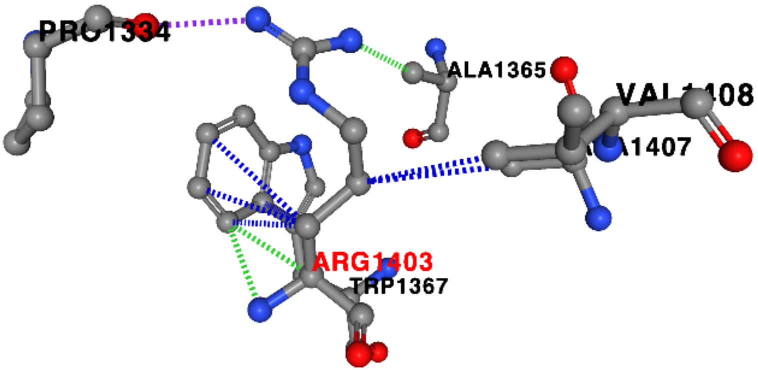

Legend:

Hydrogen Bond
Hydrophobic
Aromatic
Carbonyl
Polar
Clash
Ionic
VDW
