# Supplementary material for: Bio-Chemoinformatics-Driven Analysis of nsp7 and nsp8 Mutations and Their Effects on Viral Replication Protein Complex Stability
Source: Curr Issues Mol Biol. 2024 Mar 18;46(3):2598–619. doi: 10.3390/cimb46030165 (PMC10968879; doi:10.3390/cimb46030165)
Supplement: Supplementary file 1 [file cimb-46-00165-s001.zip › Supplementary Figure S1. Experimental Workflow.pdf]

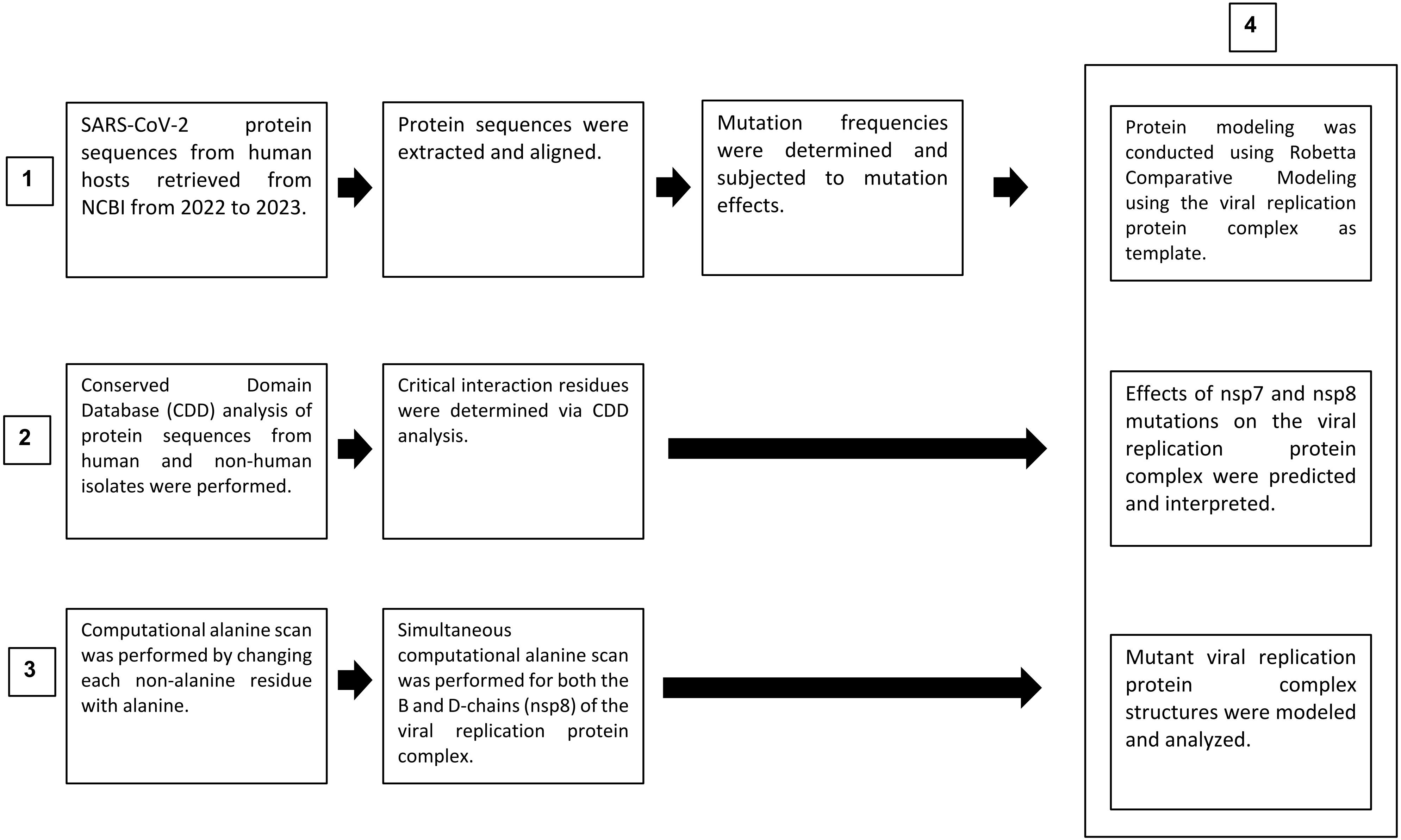

1
SARS-CoV-2 protein sequences from human hosts retrieved from NCBI from 2022 to 2023.
Protein sequences were extracted and aligned.
Mutation frequencies were determined and subjected to mutation effects.
2
Conserved Domain Database (CDD) analysis of protein sequences from human and non-human isolates were performed.
Critical interaction residues were determined via CDD analysis.
3
Computational alanine scan was performed by changing each non-alanine residue with alanine.
Simultaneous computational alanine scan was performed for both the B and D-chains (nsp8) of the viral replication protein complex.
4
Protein modeling was conducted using Robetta Comparative Modeling using the viral replication protein complex as template.
Effects of nsp7 and nsp8 mutations on the viral replication protein complex were predicted and interpreted.
Mutant viral replication protein complex structures were modeled and analyzed.

Supplementary Figure S1. Schematic Workflow of the experimental study. The study can be divided into four major parts. The first part is the temporal mutation of nsp7 and nsp8 for 2022-2023 from global sequence data. The second part is the conserved domain database analysis which identified critical interaction residues. The third part is the computational alanine scan of the viral replication protein complex. The first three parts are then subjected to the fourth part of the study which is on protein modeling and mutational effects prediction.
