# Supplementary material for: Bio-Chemoinformatics-Driven Analysis of nsp7 and nsp8 Mutations and Their Effects on Viral Replication Protein Complex Stability
Source: Curr Issues Mol Biol. 2024 Mar 18;46(3):2598–619. doi: 10.3390/cimb46030165 (PMC10968879; doi:10.3390/cimb46030165)
Supplement: Supplementary file 1 [file cimb-46-00165-s001.zip › Supplementary Figure S2. Multiple sequence alignment.pdf]

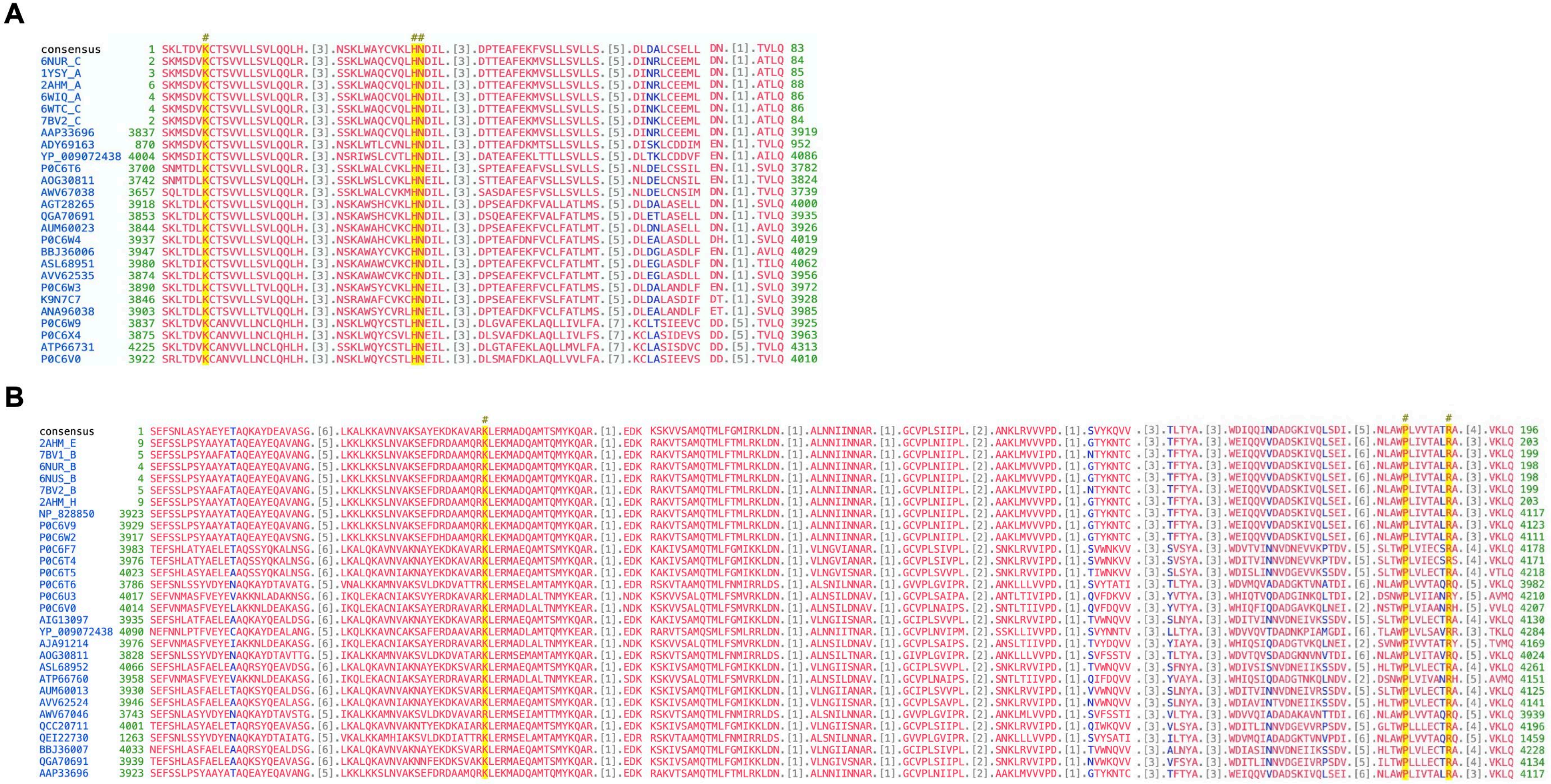

Supplementary Figure S2. Multiple sequence alignment for nsp7 (A) and nsp8 (B) during conserved domain database (CDD) analysis. The respective alignments from CDD consisted of 27 nsp7 protein sequences and their homologues and 30 nsp8 protein sequences and their homologues across different species. In nsp7, three critical interaction residues are identified: Lys-7, His-36, and Asn-37. In nsp8, the three critical interaction residues are: Lys-58, Pro-183, and Arg-190.
